# Systematic review and meta-analysis of differentially expressed miRNAs in experimental and human temporal lobe epilepsy

**DOI:** 10.1038/s41598-017-11510-8

**Published:** 2017-09-14

**Authors:** A. Korotkov, J. D. Mills, J. A. Gorter, E. A. van Vliet, E. Aronica

**Affiliations:** 1 Department of (Neuro)Pathology, Academic Medical Center, University of Amsterdam, Amsterdam, The Netherlands; 2 Center for Neuroscience, Swammerdam Institute for Life Sciences, University of Amsterdam, Amsterdam, The Netherlands; 3 Stichting Epilepsie Instellingen Nederland (SEIN), Heemstede, The Netherlands

## Abstract

Temporal lobe epilepsy (TLE) is a common chronic neurological disease in humans. A number of studies have demonstrated differential expression of miRNAs in the hippocampus of humans with TLE and in animal models of experimental epilepsy. However, the dissimilarities in experimental design have led to largely discordant results across these studies. Thus, a comprehensive comparison is required in order to better characterize miRNA profiles obtained in various post-status epilepticus (SE) models. We therefore created a database and performed a meta-analysis of differentially expressed miRNAs across 3 post-SE models of epileptogenesis (electrical stimulation, pilocarpine and kainic acid) and human TLE with hippocampal sclerosis (TLE-HS). The database includes data from 11 animal post-SE studies and 3 human TLE-HS studies. A total of 378 differentially expressed miRNAs were collected (274 up-regulated and 198 down-regulated) and analyzed with respect to the post-SE model, time point and animal species. We applied the novel robust rank aggregation method to identify consistently differentially expressed miRNAs across the profiles. It highlighted common and unique miRNAs at different stages of epileptogenesis. The pathway analysis revealed involvement of these miRNAs in key pathogenic pathways underlying epileptogenesis, including inflammation, gliosis and deregulation of the extracellular matrix.

## Introduction

Epilepsy is a common neurological disease characterized by recurrent seizures. The complex of epilepsy-associated comorbidities is a major burden for health care systems all over the world. Temporal lobe epilepsy (TLE) is one of the most common forms of focal epilepsy in adults^1, 2^. The contribution of pathological changes within the brain to the process of epileptogenesis in TLE cannot be easily addressed in human hippocampal specimens, since brain tissue is usually resected at the end-stage of the disease. Hence, a variety of experimental models have been developed to study epileptogenesis. The most widely used models that resemble the features of TLE, including hippocampal pathology, are the post-status epilepticus (SE) rodent models in which epilepsy develops after a chemically or electrically-induced SE^3^. The large-scale transcriptomic studies of such animal models have identified changes in the expression of hundreds of genes shortly after SE and during the course of epileptogenesis^4–6^. These changes have been found to underlie major pathological processes associated with human TLE, including the occurrence of spontaneous recurrent seizures, neuronal loss, aberrant growth and neurogenesis in the hippocampus, gliosis, neuroinflammation, reorganization of the extracellular matrix (ECM) and blood–brain barrier (BBB) dysfunction^7–10^.

The regulation of gene expression is not only confined to the transcriptional level. miRNAs are small non-coding RNAs that are crucially involved in the post-transcriptional control of gene expression. miRNAs act in association with the RNA-induced silencing complex (RISC), predominantly through binding to the 3′-untranslated regions (UTR) of the mature messenger RNA (mRNA) transcripts, which leads to repression of a target gene translation^11^. miRNAs have been shown to be involved in the regulation of numerous biological processes within the central nervous system^12, 13^. Differential expression of miRNAs in the temporal lobe of TLE patients and animals during the course of experimental epileptogenesis has been extensively studied over the past decade^14–18^. A variety of techniques including microarray, qPCR-array and RNA-sequencing have been utilized to profile the changes of miRNA expression in the rodent brain following the induction of SE evoked by electrical stimulation (ES)^19–23^, administration of pilocarpine^21, 24–29^ or kainic acid (KA)^30–32^. These studies have produced vast amounts of data, but their outcomes are often discordant, with each study producing a unique array of differentially expressed miRNAs.

Obtaining a clear profile of miRNA changes in animal post-SE models is confounded by many dissimilarities in experimental approaches. There are fundamental differences between models, which are characterized by different SE-triggering events and the subsequent induction of multiple pathological pathways, associated with unique patterns of transcriptional activation and miRNA deregulation. This is further complicated by differential miRNA expression between various brain regions and cell types^19, 33–35^, suggesting that the source and quality of sample preparation are important factors for miRNA profiling studies in the brain. Furthermore, the miRNA expression patterns alter dramatically over the time course of epileptogenesis^19^. Finally, technical dissimilarities in the study design, such as the choice of profiling platform, sample size, RNA extraction method, application of empirical cut-off thresholds and different statistical tests influence the study outcomes strongly.

Given its complexity, the field of epilepsy lacks the level of standardization necessary to address the role of miRNA regulation in the pathology. In the present study we integrate the existing large-scale profiling knowledge on differential miRNA expression in epilepsy, produced by the efforts of several laboratories, to create a database of differentially expressed miRNAs. We review and standardize various parameters that are crucial for comparison of profiling studies. Using a meta-analysis approach, we seek to identify consistently differentially expressed miRNAs across different studies. Such an approach has been successfully used for inter-study comparisons in cancer^36, 37^ and neurological diseases^38^. Finally, we compare biological pathways associated with altered miRNA expression in post-SE models during the chronic phase (when animals have spontaneous recurrent seizures) and human TLE-HS profiles.

## Results

### Overview

**Figure 1:**
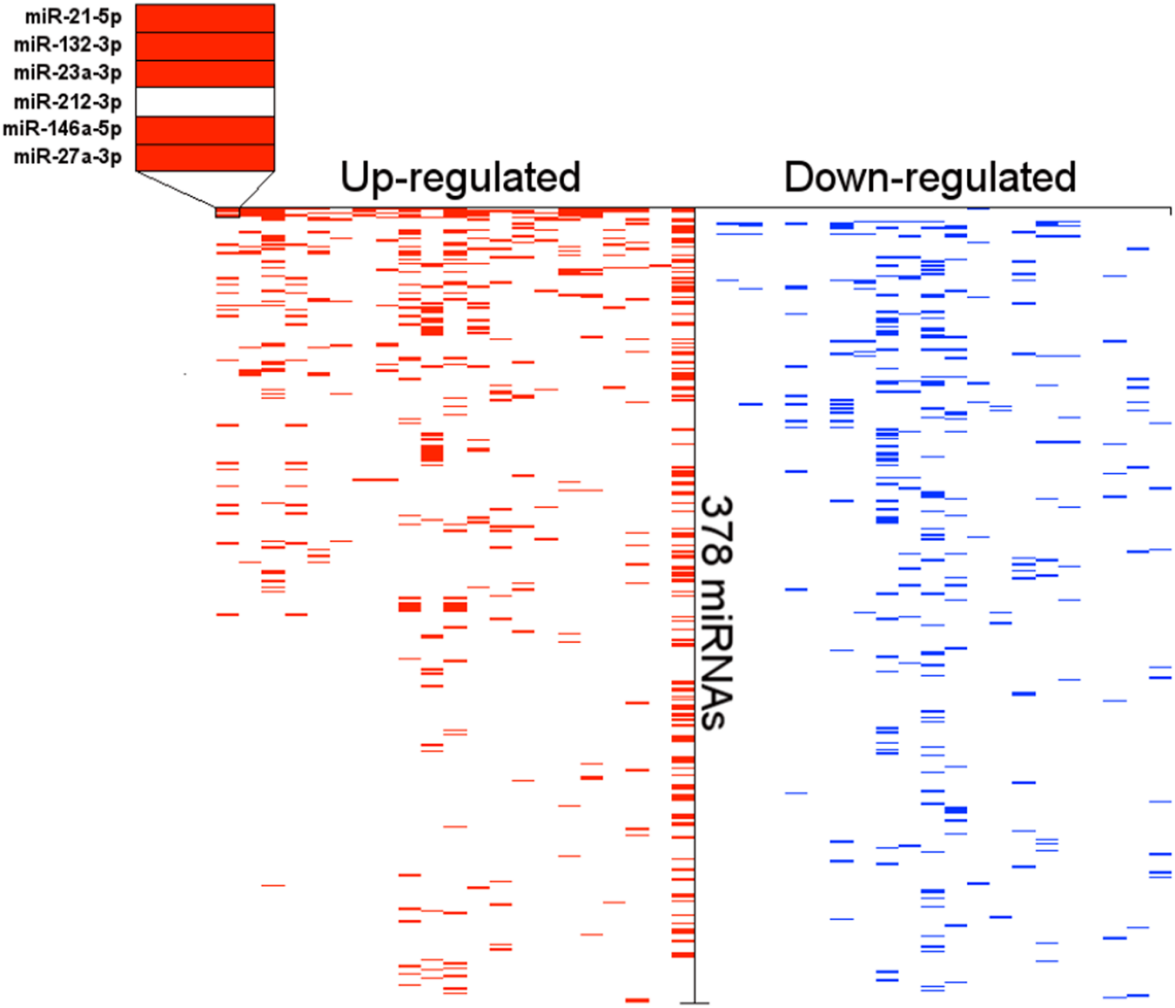
The distribution of miRNAs assorted by the frequency they appeared to be found as differentially expressed across profiling studies. The Y-axis corresponds to differentially expressed miRNAs and the X-axis corresponds to different profiles. The heatmap demonstrates that only a small proportion of miRNAs was repeatedly found to be differentially expressed and for the majority of miRNAs the differential expression was found irregularly across different profiling studies. Refer to Supplementary Table S4 for more detailed information.

The data were collected from 11 array-based and 2 RNA-Seq profiling studies in post-SE rodent models, (Table 1, Supplementary Table S1A *)* and 3 profiling studies in human TLE-HS (Table 1, Supplementary Table S1B). Using this data a database of differentially expressed miRNAs was created (Supplementary Table S2A,B *)*. The RNA-Seq studies were included in the database, however, due to the limited number of studies they were excluded from any subsequent analyses. From the 11 post-SE array-based studies 21 distinct miRNA gene expression profiles were extracted based on different animal species, post-SE models and time points. In total 378 miRNAs were found to be differentially expressed in at least one study. The expression of 274 miRNAs was increased (p < 0.05) and the expression of 198 miRNAs decreased (p < 0.05) as compared to control in at least one study. About 48% of all miRNAs (182) were found to be differentially expressed only in a single profile. Only the minority of all miRNAs were repeatedly found as differentially expressed across all profiles (Fig. 1). The most common up-regulated miRNAs across the analyzed set of expression profiles were miR-21-5p (15 profiles), followed by miR-132-3p, miR-23a-3p, miR-212-3p, miR-146a-5p, miR-27a-3p, miR-129-5p, miR-203a-3p, miR-17-5p, miR-19a-3p (Supplementary Table S4). The most common down-regulated miRNAs were miR-30a-5p (6 profiles), followed by miR-139-5p, miR-187-3p, miR-551b-3p, miR-140-3p, miR-324-5p, miR-33-5p, miR-218-5p, miR-378a-3p and miR-29c-5p (Supplementary Table S4).

**Table 1:** Included studies. miRNA profiling studies in animal post-SE models and in human TLE-HS that passed the criteria for the inclusion in the meta-analysis.

**Study**	**SE model**	**Species**	**Time points**	**Region**
Liu *et al*.^30^	KA	Rats	24 h	HP
Hu *et al*.^24^	PILO	Rats	24 h	HP
Song *et al*.^26^	PILO	Rats	60 d	HP
Jimenez-Mateos *et al*.^31^	KA	Mice	24 h	CA3
Hu *et al*.^28^	PILO	Rats	60 d	HP
Bot *et al*.^20^	ES	Rats	7d, 30 d	DG
Sun *et al*.^22^	ES	Rats	24 h	HP
Li *et al*.^23^	ES	Rats	60 d	HP
Gorter *et al*.^19^	ES	Rats	24 h, 7 d, 3–4 m	DG, CA1*
Krestschmann *et al*.^21^	ES	Mice	24 h, 28 d	HP
Krestschmann *et al*.^21^	PILO	Mice	24 h, 28 d	HP
Schouten *et al*.^32^	KA	Mice	72 h	DG
Roncon *et al*.^27^	PILO	Rats	4 d + 8 d, 11 d, 50 d	GCL
Lee *et al*.^29^	PILO	Mice	60 d	HP
Kan *et al*.^64^	TLE-HS	HP	N/A	N/A
McKiernan *et al*.^67^	TLE-HS	HP&TC	N/A	N/A
Kaalund *et al*.^65^	TLE-HS	HP	N/A	N/A

The SE in animal models was induced by chemoconvulsants kainic acid (KA) and pilocarpine (PILO) or by electrical stimulation (ES) of selected brain area (HP – hippocampus, DG – dentate gyrus, CA1/CA3 – *Cornu Ammonis*, GCL- granule cell layer of the dentate gyrus); the miRNA expression profiles were addressed at various time-points as indicated: h - hours, d - days, w - weeks, m – months following SE; the brain region used in the study is indicated as follows: HP - hippocampus, TC - temporal cortex; * - the data on the parahippocampal cortex from this study was not included. Detailed information about included studies is contained in Supplementary Table S1A,B.

### Cluster analysis

We performed an unsupervised hierarchical cluster analysis (asymmetric binary similarity measure) to estimate how well the miRNA profiles grouped by various parameters, such as model (ES, KA, pilocarpine), time point after SE and species (human TLE-HS profiles were also included for comparison). No clear clustering by any parameter was found for both up-regulation and down-regulation sets of profiles *(*Supplementary Fig. S1A,B *)*. It showed that different studies produced vastly different expression profiles. Then we extended the analysis to identify the clusters of miRNAs specific for each parameter. In order to do so, we analyzed only the miRNAs that appeared in a single model or a single time point. Several clusters of miRNAs were specific for each model with the largest cluster corresponding to ES model *(*Supplementary Fig. S2A,B *)*. The largest cluster of miRNAs specific for a particular time point corresponded to the acute stage (Supplementary Fig. S2C,D). The cluster analysis revealed some model-specific and stage-specific clusters of miRNAs with an overall high degree of heterogeneity in miRNA profiles across various studies.

### Differential expression of miRNAs across post-SE models

**Figure 2:**
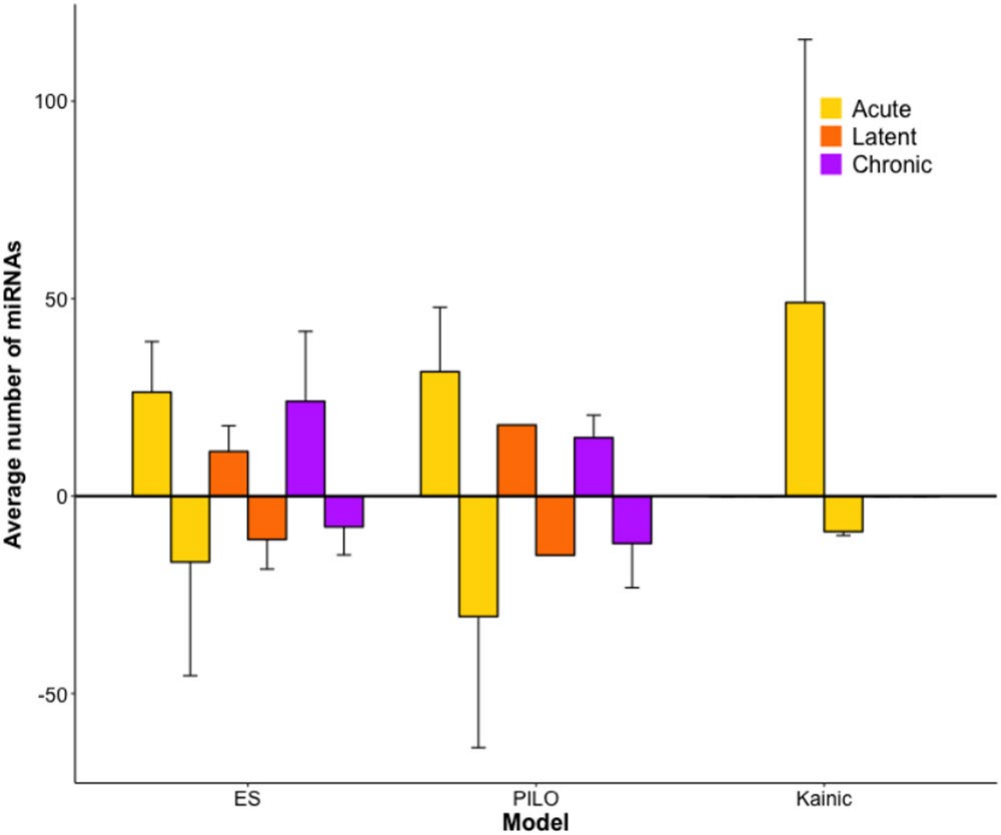
The average number of differentially expressed miRNAs produced by different sets of expression profiles. At the acute stage, the ES model had 26 ± 13 up-regulated and 17 ± 29 down-regulated miRNAs, the pilocarpine model had 32 ± 16 miRNAs up-regulated and 31 ± 33 down-regulated miRNAs, the KA model had 49 ± 67 up-regulated miRNAs and 9 ± 1 down-regulated miRNAs. At the latent stage, the ES model had 11 ± 6 up-regulated miRNAs and 11 ± 8 down-regulated miRNAs, the pilocarpine model had 18 up-regulated miRNAs and 15 down-regulated miRNAs. At the chronic stage, the ES model produced 24 ± 18 up-regulated miRNAs and 8 ± 7 down-regulated miRNAs, the pilocarpine model produced 15 ± 6 up-regulated miRNAs and 12 ± 11 down-regulated miRNAs. The KA model had a pronounced trend toward up-regulated miRNAs rather than down-regulated. The data on differential miRNA expression during the latent stage in the pilocarpine model was provided by a single profile. The standard deviation indicated large variation in the number of differentially expressed miRNAs depending on study, especially for the acute stage.

We further assessed the differences and commonalities of differentially expressed miRNAs across post-SE models. The number of differentially expressed miRNAs was not uniform both between studies, in which the same post-SE model was used and between studies of different post-SE models (Fig. 2, Supplementary Table S5). For the ES model, more up-regulated than down-regulated miRNAs were found on average at the acute and chronic stages. For the pilocarpine model, the average number of up-regulated and down-regulated miRNAs was similar. The KA model was represented only by the acute stage and produced on average the largest number of up-regulated miRNAs, however, the number of down-regulated miRNAs was the lowest amongst the acute stage profiles. The standard deviation was high for most expression sets, sometimes exceeding the average number of miRNAs. This showed that the number of miRNAs reported as differentially expressed differed greatly between studies. The ES model showed the lowest variability in the number of identified up-regulated miRNAs at the acute stage, but the pilocarpine model demonstrated better consistency in the number of miRNAs identified at the chronic stage.

**Figure 3:**
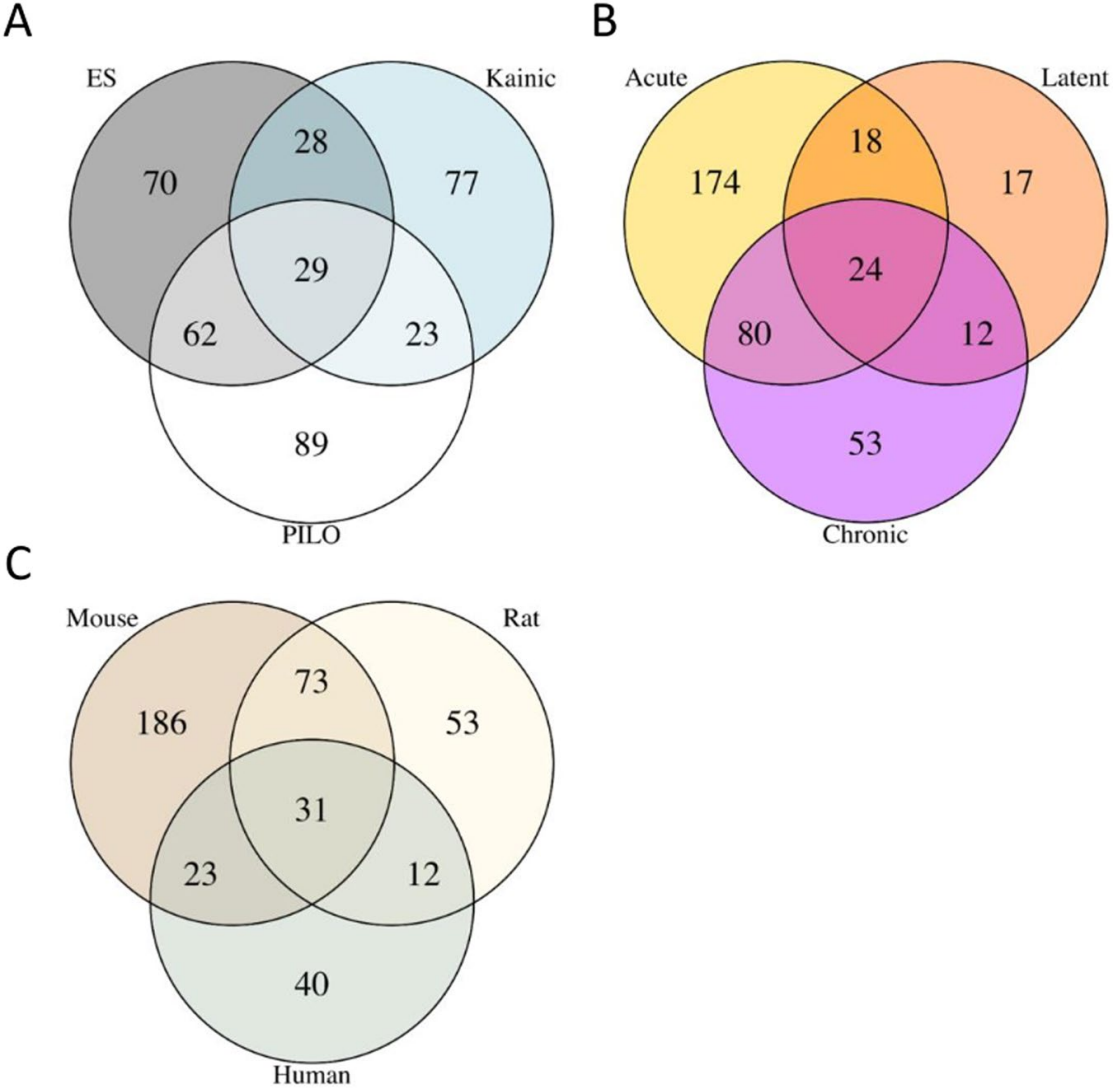
The overlap between sets of differentially expressed miRNAs. Venn diagrams demonstrate a number of common and specific differentially expressed miRNAs across different SE models (**A**), stages of epileptogenesis (**B**) and biological species (**C**). Less than 10% of the total number of identified differentially expressed miRNAs was found to overlap between all sets in each case. Many miRNAs were found only in a particular model, time point or organism.

Next, all miRNAs reported as differentially expressed in each animal model were overlapped (Fig. 3A). In total 189 miRNAs were found to be differentially expressed in the ES model, 203 in the pilocarpine model and 157 in the KA model. A large number of miRNAs was represented only by a single model: 70 for the ES, 89 for the pilocarpine and 77 for the KA. Only 29 miRNAs were shared between all 3 models together. The greatest overlap was found between the ES and the pilocarpine model. The lists of miRNAs for each model are illustrated in Supplementary Table S6A.

### Differential expression of miRNAs across stages of epileptogenesis

Next, we assessed the differences between expression profiles depending on time point following SE, but regardless of model and species. The largest number of differentially expressed miRNAs belonged to the acute stage (296), which was almost double the number of miRNAs seen at the chronic stage (169) and 4 times the amount at the latent stage (71) (Fig. 3B). There was a considerable number of miRNAs found only at a certain stage. For the acute stage this number was 174, accounting for more than 50% of all differentially expressed miRNAs at this time point. A lesser amount of miRNAs specific only for the chronic stage was identified (31%), followed by 24% for the latent stage. The overlap between all 3 stages was 24 miRNAs, only about 6% of all differentially expressed miRNAs, suggesting a dynamic regulation of miRNA expression at different stages of epileptogenesis. Supplementary Table S6B demonstrates the lists of miRNAs for each stage.

### Differential expression of miRNAs across species

Next, the differential expression of miRNAs was assessed across different species, but regardless of post-SE model or time point. We combined miRNA expression data from 7 rat and 5 mouse studies at any stage following SE and compared it to the data obtained from 3 human TLE-HS profiles (Fig. 3C). The number of differentially expressed miRNAs reported in mouse models was 313 and exceeded by far both rat (169) and human (106) studies. About 60% of all miRNAs identified in mice were not reported as differentially expressed in either rats or humans. This number was less for the rat (31%) and human (38%) datasets. As expected, the largest overlap in absolute amount of shared miRNAs was between mouse and rat species. The overlap between all three species was only 31 miRNAs, which accounts for less than 30% of all differentially expressed miRNAs in human TLE-HS. Thus, the comparison of human data with rodent data revealed that a large portion of miRNAs that were found to be differentially expressed in human TLE-HS specimens was not represented by any of the animal post-SE miRNA profiles. Supplementary Table S6C demonstrates the lists of miRNAs for each species.

We further investigated the representative value of miRNA profiles obtained in animals in regard to human TLE-HS by comparing human and rodent miRNA profiles with respect to different models and stages of SE. The human set shared approximately equal portions of miRNAs with each different model (Supplementary Fig. S3A *;* Supplementary Table S6D). Of note, the overlap between the TLE-HS and chronic post-SE profiles was about 30% *(*Supplementary Fig. S3B, Supplementary Table S6E *)*. Using a Fisher’s exact test we assessed the human TLE-HS profile for an enrichment of miRNAs from a particular stage or model. No significant enrichment for any stage or models was seen amongst human TLE-HS miRNAs (Fisher’s exact test, Bonferroni corrected p-value > 0.3).

### Identification of consistently differentially expressed miRNAs

By overlapping the differentially expressed miRNAs across different post-SE models, time points and species, largely discordant results were produced, thus a more robust statistical method known as the robust rank aggregation (RRA) method was applied to identify consistently differentially expressed miRNAs in similar studies^36, 39^. The data on fold change of differential expression and associated p-values were collected from the profiles *(*Supplementary Table S3 *)*. We performed the analysis for the data from the post-SE profiles at 3 stages. The human datasets were not used in this analysis. For the acute stage, 9 mature miRNA sequences were identified as consistently differentially expressed: 8 were up-regulated (miR-132-3p, miR-21-5p, miR-21-3p, miR-212-3p, 2137, miR-711, miR-882 and miR-142-5p) and one was down-regulated (miR-302b-5p) (Table 2). For the latent stage, 18 consistently differentially expressed mature miRNA sequences were identified: 8 were up-regulated (miR-212-3p, miR-21-5p, miR-132-3p, miR-20a-5p, miR-17-5p, miR-27a-3p, miR-23a-3p, miR-146a-5p) and 10 were down-regulated (miR-139-5p, miR-551b-3p, miR-33-5p, miR-708-5p, miR-7a-5p, miR-935, miR-138-5p, miR-187-3p, miR-30e-3p, miR-222-3p) (Table 2). For the chronic stage, 9 mature miRNA sequences were identified: 8 were up-regulated (miR-146a-5p, miR-23a-3p, miR-135b-5p, miR-21-5p, miR-132-5p, miR-132-3p, miR-210-3p, and miR-212-5p) and one was down-regulated (miR-551b-3p) (Table 2). Thus, more up-regulated than down-regulated miRNAs were found at the acute and chronic stages, but not at the latent stage which was particularly rich in down-regulated miRNAs. Noteworthy, miR-21 appeared in all 3 stages, as well as miRNAs from miR-212/132 cluster. Additionally, miR-146a-5p and miR-23a-3p were common up-regulated miRNAs for the latent and chronic stages. Among down-regulated miRNAs miR-551b-3p was common for the latent and chronic stages.

**Table 2:** Consistently differentially expressed miRNAs across all expression profiles.

**ACUTE STAGE**					
**up-regulated**			**down-regulated**		
**prefix**	**miRNA**	**p-value**	**prefix**	**miRNA**	**p-value**
h,r,m	132–3p	2.5E-07	h,m	302b-5p	1.6E-02
h,r,m	21-5p	1.4E-05			
h,r,m	21-3p	1.5E-03			
h,r,m	212-3p	2.4E-03			
m	2137	3.9E-03			
h,r,m	711	1.5E-02			
m	882	2.7E-02			
h,r,m	142-5p	3.4E-02			
**LATENT STAGE**					
h,r,m	212-3p	2.4E-06	h,r,m	139-5p	7.7E-06
h,r,m	21-5p	1.9E-05	h,r,m	551b-3p	2.6E-03
h,r,m	132-3p	4.4E-04	h,r,m	33-5p	5.1E-03
h,r,m	20a-5p	5.5E-03	h,r,m	708-5p	6.9E-03
h,r,m	17-5p	8.5E-03	h,r,m	7a-5p	1.2E-02
h,r,m	27a-3p	1.2E-02	h,r,m	935	1.2E-02
h,r,m	23a-3p	2.7E-02	h,r,m	138-5p	1.3E-02
h,r,m	146a-5p	2.8E-02	h,r,m	187-3p	1.4E-02
			h,r,m	30e-3p	2.7E-02
			h,r,m	222-3p	2.8E-02
**CHRONIC STAGE**					
h,r,m	146a-5p	6.9E-08	h,r,m	551b-3p	5.6E-03
h,r,m	23a-3p	1.5E-04			
h,r,m	135b-5p	4.4E-04			
h,r,m	21-5p	1.7E-03			
h,r,m	132-5p	1.5E-02			
h,r,m	132-3p	3.1E-02			
h,r,m	210-3p	4.1E-02			
h,r,m	212-5p	4.8E-02			

The miRNAs reported as differentially expressed were subjected to the RRA analysis; the RRA output for miRNAs at the acute, latent and chronic stages post-SE. The analysis was done including only the miRNA signatures found in more than one study. Prefixes are abbreviated as follows: h-hsa, r-rno, m-mmu; p-value <0.05.

### Pathway analysis of consistently differentially expressed miRNAs

**Figure 4:**
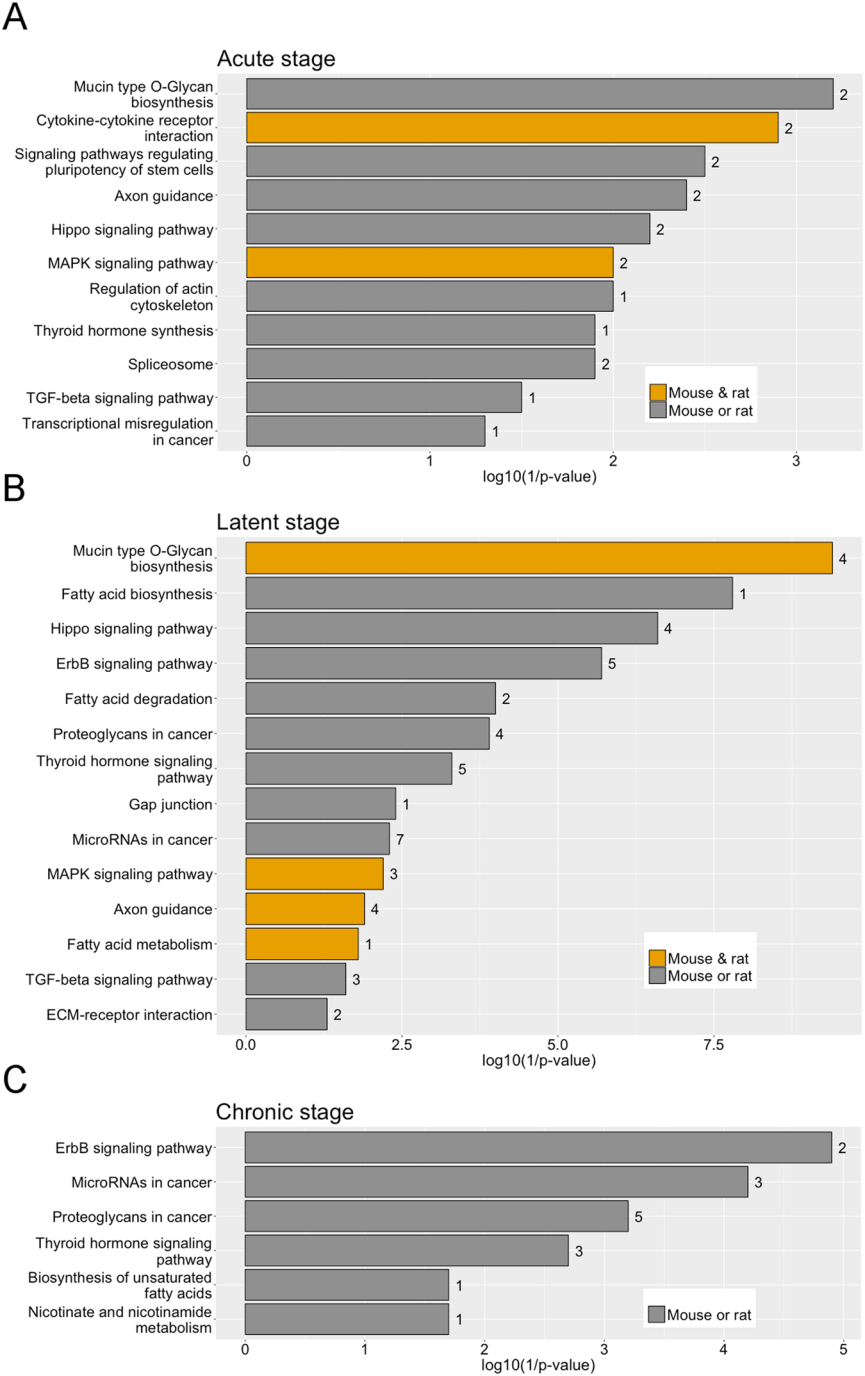
KEGG pathway enrichment analysis. The analysis was performed using DIANA miRPath ver.3 algorithm (“Pathways Union” mode) for consistently differentially expressed miRNAs, produced by RRA analysis and human TLE-HS miRNAs. The first 10 most enriched pathways for each section are presented; (**A**) pathways enriched for miRNAs at the acute stage, (**B**) pathways enriched for miRNAs at the latent stage, (**C**) pathways enriched for miRNAs at the chronic stage; non-relevant pathways were excluded. The number of miRNAs is indicated next to each bar﻿﻿; FDR < 0.05.

miRNAs identified as consistently differentially expressed at each stage of epileptogenesis were subjected to the Kyoto Encyclopedia of Genes and Genomes (KEGG) pathway analysis using Diana miRPath3. The pathway analysis was done for mouse and rat species. At the acute stage “MAPK signaling” and “Cytokine-cytokine receptor interaction” were the only common pathways for both rat and mouse species (Fig. 4A, Supplementary Fig. S4A). Among the most enriched pathways identified only in rats or mice were “Mucin type O-Glycan biosynthesis”, “Signaling pathways regulating pluripotency of stem cells” and “Axon guidance”.

The latent stage was accompanied by activation of a variety of signaling pathways and, similar to the acute stage, showed the involvement of miRNAs in the MAPK signaling (Fig. 4B, Supplementary Fig. S4B). However, this stage was marked by the stronger association of miRNAs with the ECM-related signaling pathways, including “Mucin type O-Glycan biosynthesis” and “Axon guidance”. Among the most enriched pathways identified only in rats or mice “Fatty acid biosynthesis”, “Hippo signaling”, “ErbB signaling” were prominent.

For the miRNAs at the chronic stage of epileptogenesis, common pathways for both rodent species were found only by the “Gene union” function (Supplementary Fig. S4C). “Proteoglycans in cancer” was the most enriched pathway, followed by “Ubiquitin mediated proteolysis”. The most enriched pathways identified only in rats or mice using “Pathway union” were “ErbB signaling pathway”, “Proteoglycans in cancer” and “Thyroid hormone signaling pathway” (Fig. 4C).

### Comparative pathway analysis of differentially expressed miRNAs in chronic post-SE and TLE-HS profiles

**Figure 5:**
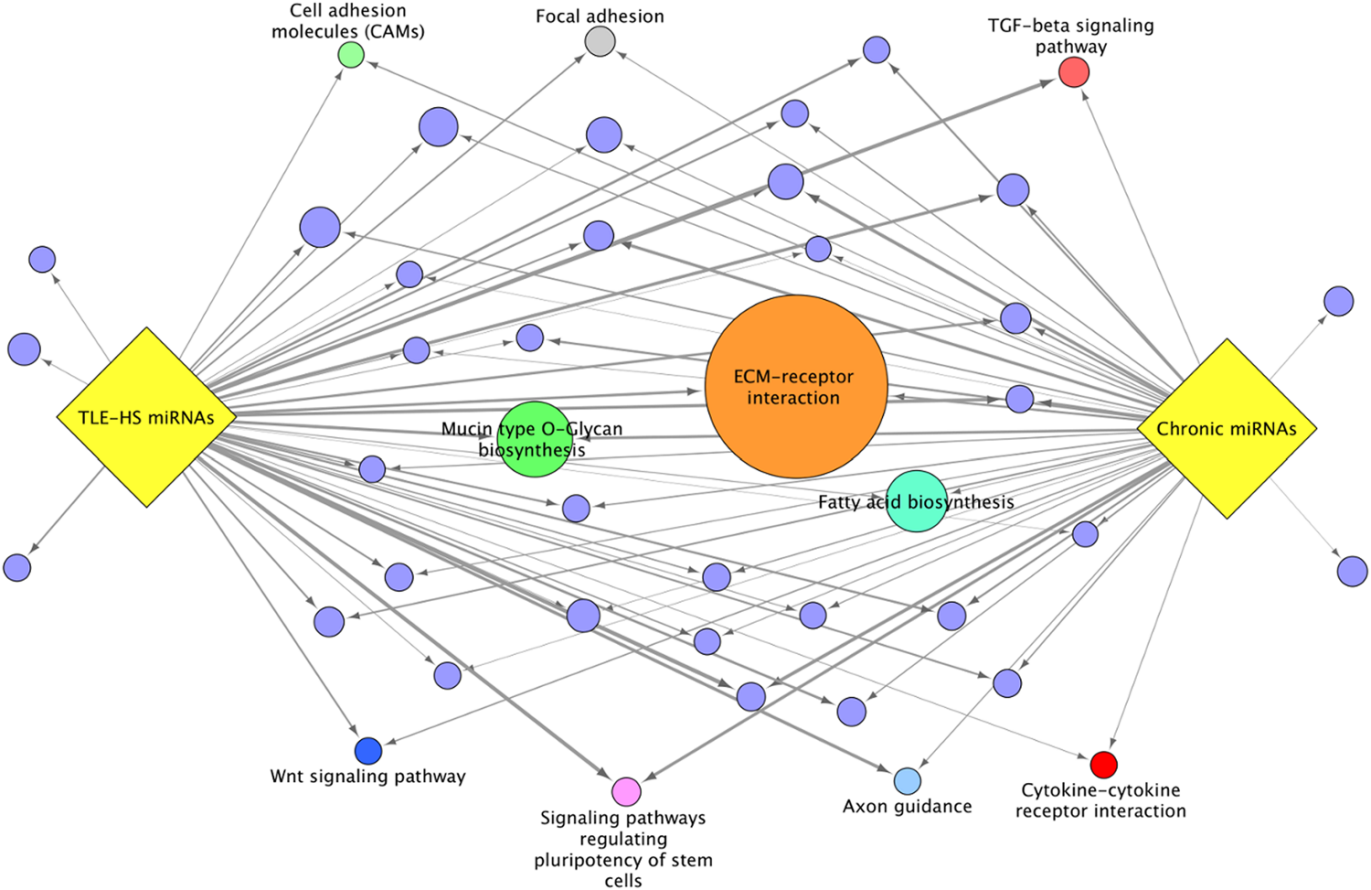
Enriched pathways from human TLE-HS and from post-SE rodent models during the chronic stage. Only the top 30 enriched pathways from human TLE-HS and post-SE rodent models are shown. The size of the node is inversely proportional to the adjusted p-value of the enriched pathway. Nodes are connected if the pathway was enriched amongst the human miRNAs or the rodent miRNAs. Informative pathways are labeled. For a complete list of the pathways please see Supplementary Table 7.

Finally, we addressed how well the biological pathways at the chronic stage correspond to the biological pathways in human TLE-HS. We performed a pathway enrichment analysis for the 100 most consistently differentially expressed miRNAs in chronic post-SE profiles and compared them to the pathways associated with the 100 most consistently differentially expressed miRNAs in the TLE-HS profile (Supplementary Table S6F). A large variety of pathways were found to be enriched for the rodent and TLE-HS sets of differentially expressed miRNAs (Supplementary Table S7A,B). The interaction map combining both datasets demonstrated a remarkable overlap between the pathways (Fig. 5). On analysis of the top 30 enriched pathways for the rodent profiles, 28 also appeared as enriched amongst the TLE-HS profiles, for the top 30 human TLE-HS profiles 27 were also enriched amongst the rodent chronic profiles. One of the largest clusters of the enriched pathways was associated with the ECM. The most significantly enriched pathways within this cluster were the “ECM-receptor interaction” and “Mucin type O-Glycan biosynthesis”. Another cluster of enriched pathways was associated with the fatty acid biosynthesis and processing. The example map of genes targeted within the “ECM-receptor interaction” pathway is displayed in Supplementary Fig. 5. The biological pathways associated with the differentially expressed miRNAs at the chronic stage post-SE were found to be representative of the pathways associated with the differentially expressed miRNAs in human TLE-HS profiles.

## Discussion

The development of tools for a large-scale evaluation of gene expression over the previous decade has allowed for the collection of extensive data on miRNA regulation in epilepsy. Many studies have attempted to create a profile of differentially expressed miRNAs using animal post-SE models. However, the heterogeneity of features carried by different experimental models, as well as by human TLE, has led to largely discordant results across these efforts. In the present study, we performed a meta-analysis across various published miRNA expression profiles and employed bioinformatical tools to investigate pathological cellular pathways involved in different phases of epileptogenesis.

The brain is exceptionally rich in the number and spatial distribution of miRNAs^40^. We created a database of more than 400 mature miRNA sequences that have been reported to be differentially expressed in post-SE rodent models of epileptogenesis and in human TLE-HS. Our analysis showed that only a small portion of these miRNAs is consistently deregulated across various profiling studies with the rest of them appearing rather occasionally. This implies that a compact core body of miRNAs involved in key pathogenic processes exists that is most strongly associated with the development of the pathology, and a very long tail of miRNAs exists that is associated with all the variety of features accompanying each particular case. In experimental epilepsy, these differences arise from the diversity in study approaches, such as the type of model and species used, the region of the brain and the time point of epileptogenesis at which miRNA expression is assessed^14, 41, 42^. This diversity of input parameters is further accompanied by different processing of samples and data^43^, followed by application of various profiling platforms and approaches for normalization and statistical analysis of data^44^. As a result of this heterogeneity of parameters, our cluster analysis revealed a poor clustering of miRNA profiles extracted from different studies. Each individual profile seems to provide a piece of a bigger picture, covering some aspects of the pathology or representing one of the possible routes of epileptogenesis.

The 3 investigated post-SE models have produced more miRNAs specific for each model than shared between the 3 models. The KA model carried the most unique set of miRNAs among the 3 models. The KA profiles had a pronounced trend toward up-regulation with a few down-regulated miRNAs detected. In addition, all KA profiles were confined to early time points representing acute changes. Many miRNAs reported as deregulated in the KA model were found to be differentially expressed only in a single study. The miRNA profiles of the pilocarpine and ES models have been previously shown to cluster closer between the acute and chronic time points within the same model than between the corresponding time points of the two models^21^. Our analysis revealed approximately equal amounts of miRNAs that were shared between the ES and pilocarpine models and that were specific for each model alone. Large clusters of miRNAs were identified as specific only to some particular model. This was not observed for the KA model, because the cluster analysis was done for the miRNAs reported in at least 2 studies, but many differentially expressed miRNAs in the KA model appeared only in a single profile, hence, were excluded from the cluster analysis. The largest clusters of model-specific miRNAs were found for the ES model. These miRNAs may be deregulated exclusively as a result of electro-stimulation, however not being captured in other models. The existence of these ES model-specific miRNAs may also be explained by the fact that 2 out of 3 ES studies investigated expression of miRNAs in the subregions of the hippocampus. For comparison, 5 out of 6 pilocarpine studies focused on the entire hippocampus rather than its subregions. Different hippocampal subfields are known to be molecularly diverse^45, 46^ with variable miRNA distribution^47^. Each cell type that belongs to a particular subfield also displays different patterns of miRNA expression^33–35^. The studies focusing on a particular subregion of the brain allow identification of specific miRNAs, however, the broad picture may be missed. The current knowledge of miRNA profiles in experimental epilepsy models is shifted towards subregion specificity in case of the ES model and towards the entire hippocampus picture in case of the pilocarpine model.

The profiles appeared to be largely different not only by post-SE models, but also by the time points following SE. The largest number of differentially expressed miRNAs was found at the acute stage (24 hours post-SE in most studies). Besides, we found a high variability in the number of differentially expressed miRNAs between the profiles at the acute stage, which may be associated with the larger entropy of changes at this time point. The ES model showed less inter-study variability in the number of up-regulated miRNAs at the acute stage compared to the two chemoconvulsant models. The use of different dosages of chemoconvulsants and the drugs for the cessation of SE may result in different courses of epileptogenesis^7, 48^ with variable amplitude and character of molecular changes in the brain, including miRNA regulation.

Despite a very small overlap between the 3 stages, the acute and chronic stages of epileptogenesis shared a considerable amount of miRNAs. The miRNA profiles at the chronic stage may be affected by the acute seizure-related changes if a period of time between the last spontaneous seizure and the sacrifice is short. It has been demonstrated that miRNA expression patterns were clearly different between the acute seizure model and the chronic model^21^. Thus a single seizure episode prior to sacrifice may significantly alter the outcome profile and that must be thoroughly controlled in order to get reproducible results.

Considering the differences between the profiles, the involvement of miRNAs in epilepsy can hardly be concluded based on the observations made in one single profile, but rather the relevance of a miRNA to some pathogenic pathways activated in a chosen model in particular sub-region of the brain and at given time point may be assessed. In order to identify consistently differentially expressed miRNAs at different stages of post-SE epileptogenesis we applied the RRA method^39^. While this method drastically reduced the number of differentially expressed miRNAs it highlighted a handful of miRNAs for further study. A relatively unique profile of miRNAs accompanied each stage. Combined, these miRNA signatures may be characteristic of the major pathogenic processes underlying epileptogenesis, such as cell death, gliosis and neuroinflammation^49–52^. The only 3 common miRNAs that were found between all 3 stages of epileptogenesis were miR-21–5p, miR-132–3p and miR-212–3p. Both miR-21 and the cluster of miR-212/132 have been implicated in epilepsy and various neurologic disorders^53, 54^. Deregulation of these miRs may represent the interplay between neuronal cell death and neuroprotection, as both miR-21 and miR-212/132 have been previously implicated in these processes in the brain^31, 55^. The role in inflammation has also been attributed to both miR-21^56^ and miR-132^57^. Our pathway analysis showed that miRNAs at the acute stage were strongly involved in the cellular pathways associated with innate immune response and inflammation. At the latent stage, up-regulated miRNAs miR-20a, miR-17, miR-23a, miR-27a and miR-146a have been previously shown to be enriched in mouse microglia^58^. Noteworthy, both the latent and chronic stages were marked by the up-regulation of miR-146a, a key regulator of inflammation in astrocytes^59^.

In addition to the observed differences in miRNA profiles between different post-SE models and time points, there were differences associated with species. We found a large amount of miRNAs that were identified only in rats, mice or humans. That raised a question of how relevant the findings in rodents are to human TLE. The comparison of the results obtained in animal post-SE profiles and in human TLE profiles is hampered by many obstacles. The amount of identified miRNAs, their predicted targets and functions may vary considerably between different species^60–62^. There are also certain limitations of using human TLE tissue, like the quality of RNA and the use of autopsy control samples, heterogeneity of epilepsy cases and limited supply^63^. Patients undergoing hippocampal resection in most cases have a long clinical history of various anti-epileptic drug therapies, altering the molecular profile. Besides, SE is rarely an initiation event for epilepsy in humans, while it is characteristic for animal post-SE models.

The profiles of miRNA expression in TLE-HS have been reported by several research groups^64–67^. Neither of TLE-HS profiles included in the meta-analysis has reported significant up-regulation of miR-21 or miR-132. However, both these miRNAs have been found to be overexpressed in human TLE-HS when their expression was assessed individually^68^. In order to identify the biological pathways associated with the differentially expressed miRNAs in human TLE-HS we combined the results of the TLE-HS profiles and subjected them to the pathway analysis taking into account the combinatorial effect of all miRNAs^69^. Since the chronic stage in post-SE models has been considered to be representative of the neuropathological features of human TLE-HS, we performed the pathway enrichment analysis for the 100 most consistently differentially expressed miRNAs in chronic post-SE profiles and compared the results with the enriched pathways associated with the 100 most consistently differentially expressed miRNAs in TLE-HS. The pathways associated with the differentially expressed miRNAs in chronic profiles and in TLE-HS largely overlapped. The most enriched pathways were related to the regulation of the ECM and fatty acids.

The ECM is an important player in the epileptogenic network and the involvement of miRNAs in neuroinflammation, reactive gliosis and BBB breakdown may be directly or indirectly associated with the ECM remodelling, which can be evident already during the latent stage following SE^4^. Consistent with our observations a recent transcriptome analysis in 129 TLE patients has revealed the cell-to-extracellular matrix adhesion processes (“ECM-receptor interaction”, “focal adhesion”) as one of the two major clusters of differentially expressed genes along with the inflammation^70^. The changes in expression of the ECM genes cause alterations in the molecular environment surrounding neurons, which is a hallmark of epileptogenesis^4^. The components of the brain ECM produced by both neurons and glial cells are involved in multiple physiologic processes, which are disturbed as a result of the ECM remodeling, leading to further epileptogenic events, such as mossy fiber sprouting, granule cell dispersion and astrogliosis^71^. Interestingly, the miRNAs associated with the “ECM-receptor interaction” pathway in our analysis were different between the chronic post-SE profiles and the TLE-HS profiles. These observations suggest that deregulated miRNAs and their target genes may differ between the rodent and human species, but the pathogenic pathways, that the deregulated miRNAs are associated with, are generally conserved across species and can be indicative of the TLE neuropathological features. Thus, the deregulated pathways, rather than the individual miRNAs may be of more interest when translating results from rodent models to TLE. Of note, there was little overlap in identified differentially expressed miRNAs across the 3 TLE-HS profiles and we acknowledge that the low consistency across these studies does not allow a proper statistical evaluation of these data. Thus, there is a pressing need for more profiling studies on differentially expressed miRNAs in TLE-HS, which will allow a better characterization of the translational validity of miRNA expression data from rodents to humans.

Currently, the majority of miRNA profiling in post-SE rodent models and human TLE have utilized microarrays or qPCR arrays. Moving forward high-throughput RNA-Seq will become the standard technique used for the identification of differentially expressed miRNAs. RNA-Seq offers a relatively unbiased, discrete, and digitized read count from a cDNA library input. When compared to microarrays, RNA-Seq has lower levels of background noise, produces less false positives, has a higher dynamic range, requires a lower amount of input RNA and can be used to detect novel short non-coding RNA species and isomiRs^72–75^. Further, the differential expression of miRNAs in a microarray experiment is defined by the fold change of expression. The fold change may be low if a miRNA is highly expressed at basal level and may be high if the miRNA is not abundantly expressed. Thus, the fold change value will be practically meaningless for the low-expressed miRNAs, while the changes in highly expressed miRNAs will be missed if a fold change cut off is applied^76^. This issue is somewhat alleviated in RNA-Seq, as an RNA-Seq output can provide a quantitative expression output, such as raw or normalized read count or counts per million (CPM) for each miRNA. This can allow lowly expressed miRNAs to be filtered out, or for an exploration of the nature of the changes in expression. Overall, RNA-Seq offers a higher resolution, more comprehensive view of the small RNA transcriptome that will not only increase confidence in the role of previously identified miRNAs in epileptogenesis but also potentially identify new key players.

In this work we combined data from various profiling studies and created a database of differentially expressed miRNAs in epilepsy. The analysis of these data has demonstrated that the outcomes of different profiling studies are vastly different due to heterogeneity of input parameters. The profiles differ depending on the type of post-SE model, time point, region of the brain and species. Despite many differences between the profiles we identified the most common differentially expressed miRNAs across the studies. For each stage of epileptogenesis there was a certain combination of consistently differentially expressed miRNAs, associated with the key pathogenic pathways underlying such processes as neuroinflammation, gliosis, cell death and deregulation of the ECM. We identified only the most consistently differentially expressed miRNAs, but many model- and process- specific miRNAs should exist that are associated with all the variety of biological processes. The major pathways, associated with deregulated miRNAs in post-SE models may be conserved across mammalian species and be characteristic of epileptogenesis in human TLE. The analysis of the pathways associated with the differentially expressed miRNAs has revealed a remarkable similarity between the human TLE-HS and chronic profiles in post-SE rodent models with a cluster of the ECM-related pathways among the most enriched. Thus, a better understanding of miRNA regulation during the course of epileptogenesis in rodent models has the ability to contribute to the development of preventive treatment for TLE.

## Methods

### Collection of meta-data

The studies containing profiles of miRNA expression in rodent models of epileptogenesis and in human TLE with hippocampal sclerosis (TLE-HS) were searched through PubMed database using the keywords “miRNA”, “epilepsy”, “status epilepticus”, “profile”, “micro-array” in various combinations. The last search was performed in May 2017. The content of each study was carefully analyzed. Only studies published in peer-reviewed journals were included in the analysis. Table 1 contains the list of included studies with a brief description (Table 1). The detailed information about included studies can be found in Supplementary Table S1A,B. This includes: author, year of publication, SE model type, species and number of recruited animals, assay platform, number of profiled miRNAs, cut-off criteria, time point of miRNA expression assessment, region of brain, drug history and method of RNA extraction. The studies in animal models were included if the following criteria were met: rodent species were used (rats or mice); SE was established and the comparison of miRNA expression profiles was done between SE and an untreated control group; the hippocampal structures were assessed. The miRNA expression profiles of kindling and traumatic brain injury (TBI) models of epileptogenesis were not included in the analysis. The studies of miRNA expression in human TLE were included if the comparison was made between hippocampal sclerotic tissue obtained from TLE patients (surgical resection or autopsy) and control hippocampal tissue (autopsy). Some of the notable studies did not pass these criteria and were not included in the analysis (Supplementary Table S1C,D). Only the studies, in which miRNA array or qPCR array based techniques were used for the profiling were included in the analysis. High-throughput sequencing studies and studies of individual miRNAs using qPCR technique were not included in the analysis.

### The database of differentially expressed miRNA

We created a database of differentially expressed miRNAs in post-SE rodent models and human TLE-HS. Mature miRNA sequences were arranged in a table with respect to species (mouse or rat), anatomical region (hippocampal subregions) and time point of assessment (Supplementary Table S2A,B). The database included data on differentially expressed miRNAs from both array-based and RNA-seq-based profiles. The time points were divided into 3 groups based on the information from each study and according to the time elapsed after SE: acute stage (24–72 hours), latent stage (7–11 days), chronic stage (≥28 days). miRNAs were considered to be significantly differentially expressed according to the cut-off and p-value criteria set by the authors of each study. The fold change of differential miRNA expression and associated p-values were also collected, when provided (Supplementary Table S3). The supplementary information published online for each study was used if available and the authors were contacted directly if additional information was needed.

### Nomenclature of miRNAs

The IDs were assigned to miRNAs according to the nomenclature recommended by miRbase (http://www.mirbase.org, edition 21), however, all species indicators were abandoned to simplify the comparison of miRNA profiles between different species. The comparison of miRNAs between different species was done based on total sequence homology between orthologues. If the miRNA ID obtained from the selected studies had outdated nomenclature, it was substituted with the new one. The miRNA signatures of species other than human, rat or mouse and non-miRNA probes were excluded from the analysis.

### Robust Rank Aggregation (RRA) analysis and statistical analysis

The lists of extracted miRNAs were prioritized based on statistical test adjusted *p*-values (less than 0.05 was assumed to indicate a significant difference). The lists of extracted miRNAs from each animal post-SE study were split into up-regulated and down-regulated miRNAs. The human TLE-HS profiles were not analyzed by the RRA. The miRNAs in each list were ranked by p-value, when possible. If no p-value was available, the miRNAs were ranked by fold change value. To identify miRNAs that were ranked consistently better than expected by chance, we used the RRA method as implemented in the R package^39^. This method assigns a p-value to each element in the aggregated list indicating how much better it is ranked compared with a null model expecting random ordering. All statistical and clustering analyses were carried out using the statistical software R (www.r-project.org).

### Target pathway prediction

The miRNAs identified by the RRA analysis as consistently differentially expressed were submitted to DIANA mirPath v.3 (http://snf-515788.vm.okeanos.grnet.gr/), a web-based computational tool that identifies potentially altered molecular pathways by the expression of multiple miRNAs. This program performs an enrichment analysis of miRNA target genes, and compares each set of miRNA targets to all known KEGG pathways^77–79^. We used the microT-CDS 5.0 algorithm for target prediction. The lists of differentially expressed miRNAs were analyzed separately for each species (mouse, rat, human). The common pathways enriched for both mice and rats were united based on the geometric mean of their p-values. The non-common pathways between the rodent species were merged together and ranked based on their p-values. The pathway analysis was done using both “Gene union” and “Pathway union” algorithms^69^. The pathways were considered to be enriched if they passed the p-value threshold 0.05. All p-values were FDR-corrected.

### Data availability

All data generated or analysed during this study are included in this published article (and its Supplementary Information files).

## Electronic supplementary material

Supplementary Figures and Tables S1, S7

Supplementary Table S2

Supplementary Table S3

Supplementary Table S4

Supplementary Table S5

Supplementary Table S6
