# Supplementary material for: Systematic review and meta-analysis of differentially expressed miRNAs in experimental and human temporal lobe epilepsy
Source: Sci Rep. 2017 Sep 14;7:11592. doi: 10.1038/s41598-017-11510-8 (PMC5599629; doi:10.1038/s41598-017-11510-8)
Supplement: Supplementary file 1 — Supplementary Figures and Tables S1, S7 [file 41598_2017_11510_MOESM1_ESM.pdf]

Systematic review and meta-analysis of differentially expressed miRNAs in experimental and human temporal lobe epilepsy

A. Korotkov, J.D. Mills, J.A. Gorter, E.A. van Vliet, E. Aronica

Supplementary Figures and Tables S1, S7

A

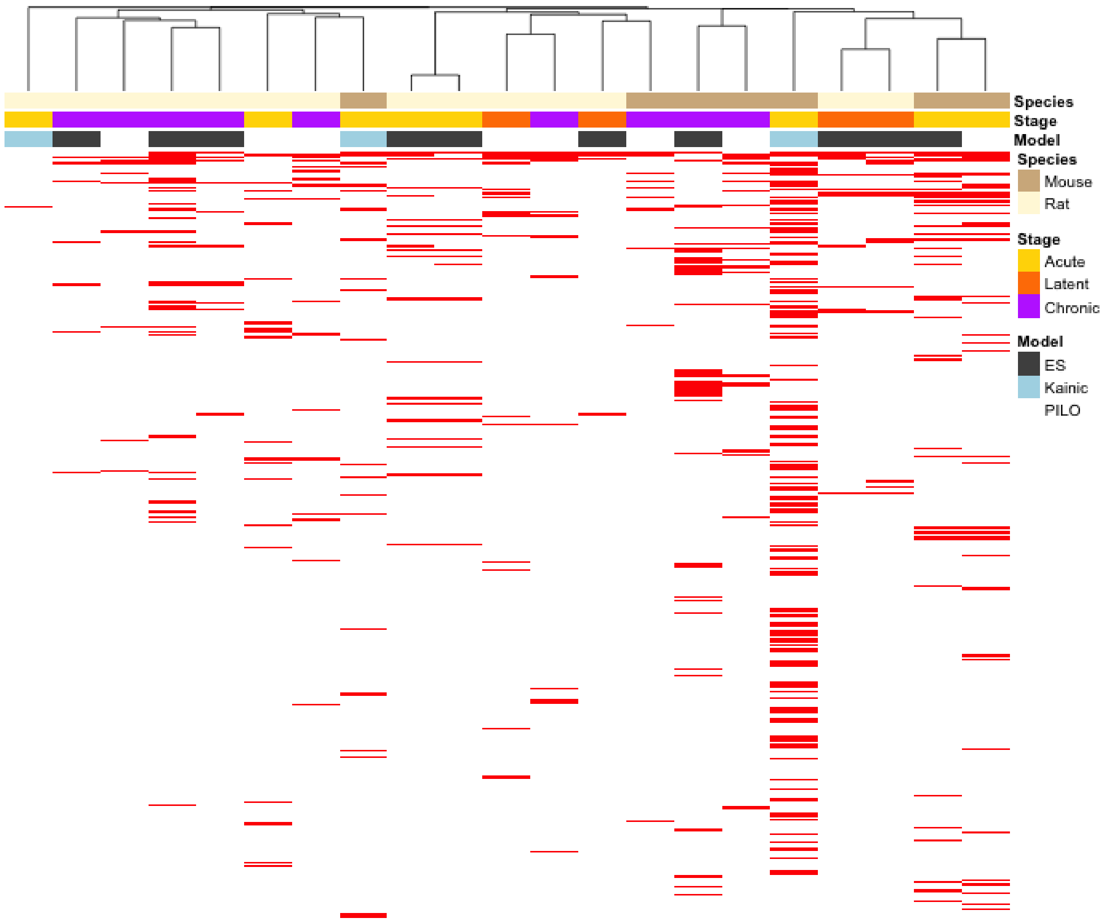

Supplementary Fig. S1: (A) - unsupervised cluster analysis of up-regulated miRNAs; (B) - unsupervised cluster analysis of down-regulated miRNAs.

B

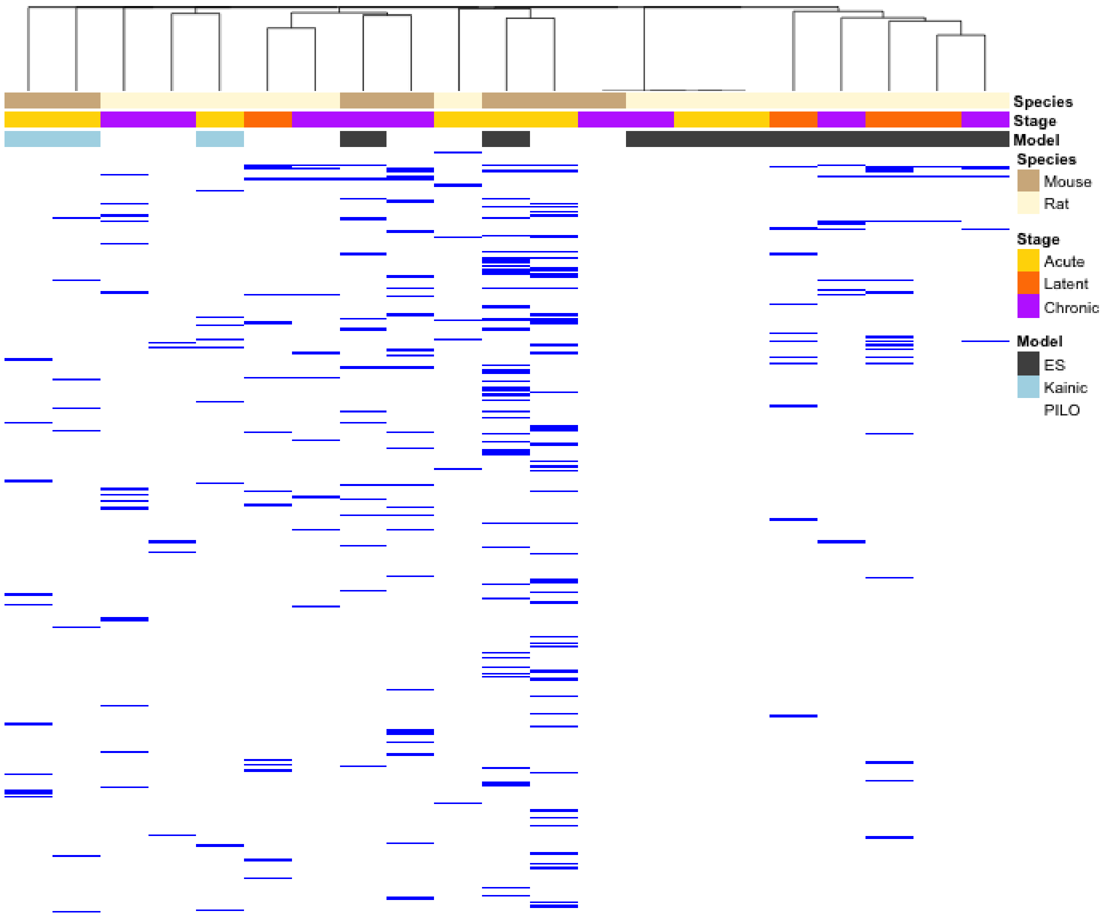

Supplementary Fig. S1: (A) - unsupervised cluster analysis of up-regulated miRNAs; (B) - unsupervised cluster analysis of down-regulated miRNAs.

A

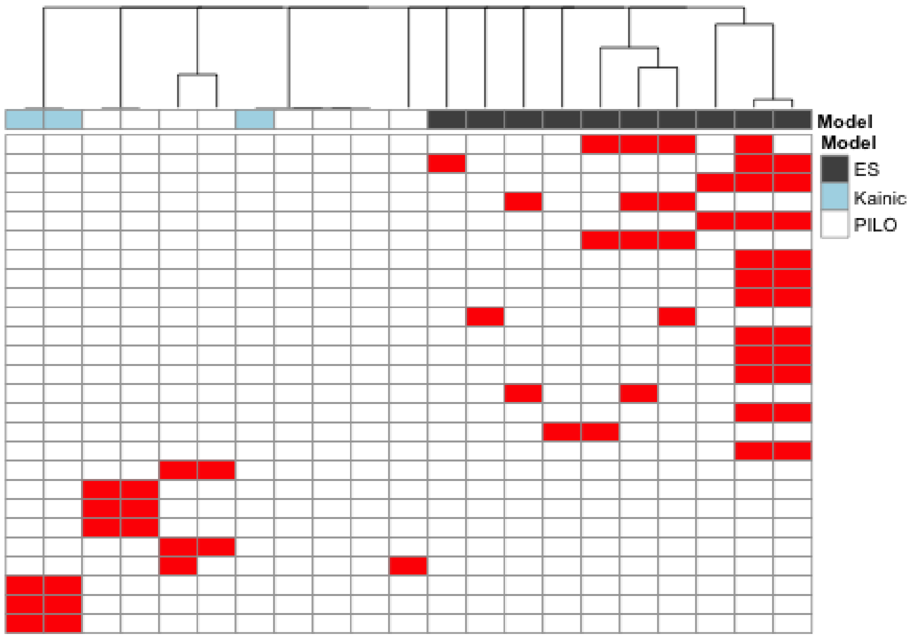

Supplementary Fig. S2: Model-specific and stage-specific clustering of differentially expressed miRNAs. miRNAs were considered model-specific if they appeared in at least 2 studies of the same model and not in any other model. miRNAs were considered stage-specific if they appeared in at least 2 studies of the same stage and not in any other stage; (A) - up-regulated, model-specific miRNAs; (B) - down-regulated, model-specific miRNAs; (C) - up-regulated, stage specific miRNAs; (D) - down-regulated, stage-specific miRNAs.

B

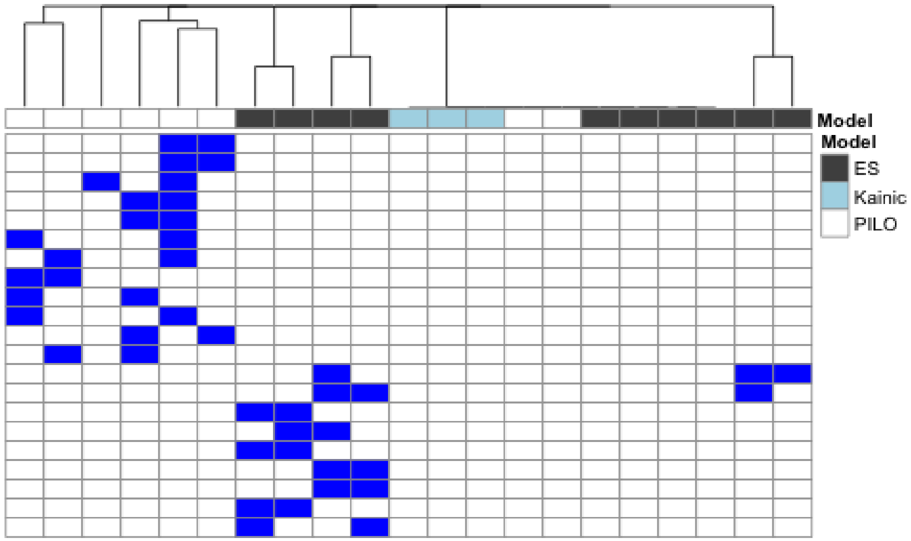

Supplementary Fig. S2: Model-specific and stage-specific clustering of differentially expressed miRNAs. miRNAs were considered model-specific if they appeared in at least 2 studies of the same model and not in any other model. miRNAs were considered stage-specific if they appeared in at least 2 studies of the same stage and not in any other stage; (A) - up-regulated, model-specific miRNAs; (B) - down-regulated, model-specific miRNAs; (C) - up-regulated, stage specific miRNAs; (D) - down-regulated, stage-specific miRNAs.

C

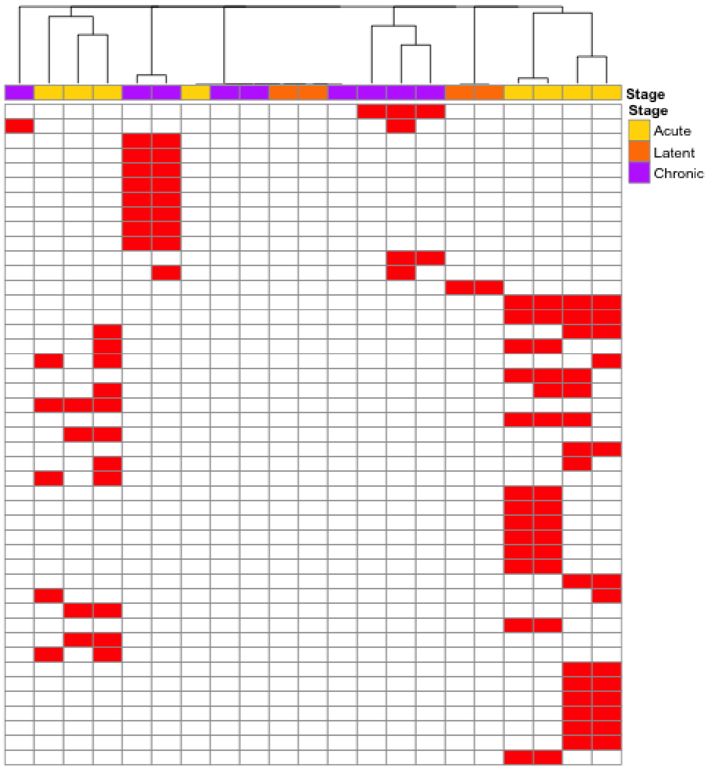

Supplementary Fig. S2: Model-specific and stage-specific clustering of differentially expressed miRNAs. miRNAs were considered model-specific if they appeared in at least 2 studies of the same model and not in any other model. miRNAs were considered stage-specific if they appeared in at least 2 studies of the same stage and not in any other stage; (A) - up-regulated, model-specific miRNAs; (B) - down-regulated, model-specific miRNAs; (C) - up-regulated, stage specific miRNAs; (D) - down-regulated, stage-specific miRNAs.

D

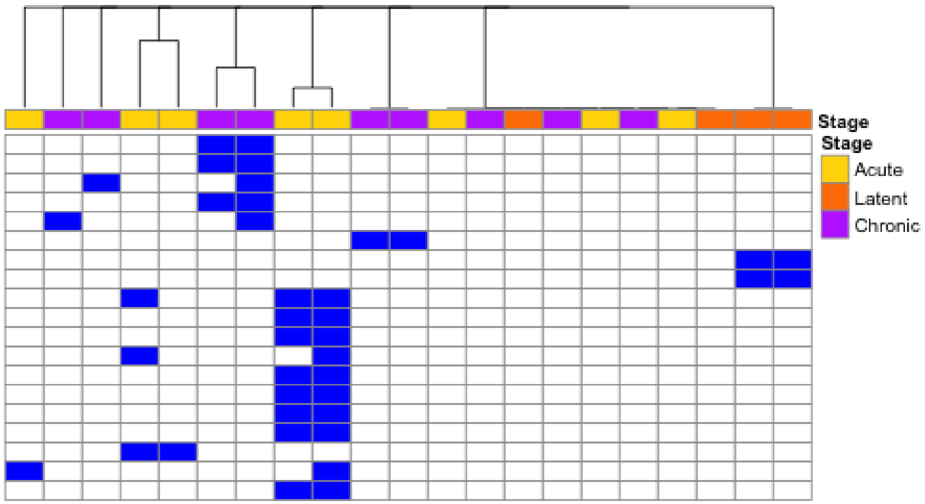

Supplementary Fig. S2: Model-specific and stage-specific clustering of differentially expressed miRNAs. miRNAs were considered model-specific if they appeared in at least 2 studies of the same model and not in any other model. miRNAs were considered stage-specific if they appeared in at least 2 studies of the same stage and not in any other stage; (A) - up-regulated, model-specific miRNAs; (B) - down-regulated, model-specific miRNAs; (C) - up-regulated, stage specific miRNAs; (D) - down-regulated, stage-specific miRNAs.

A

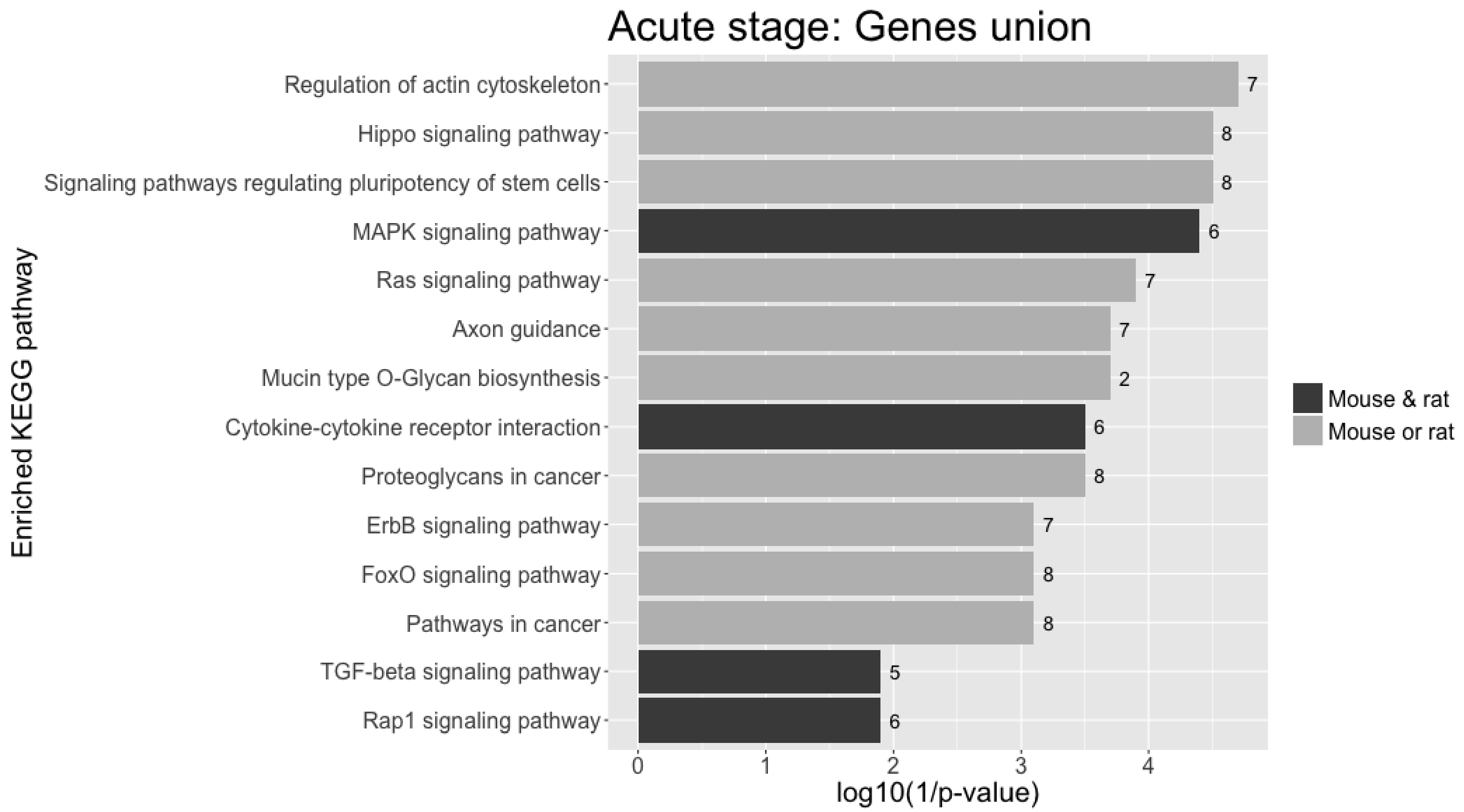

Supplementary Fig. S3: KEGG pathways enrichment analysis performed using DIANA miRPath ver.3 algorithm (Gene union mode) for consistently differentially expressed miRNAs, produced by RRA analysis and human TLE miRNAs. The first 10 most enriched pathways for each section are presented; (A) – Pathways enriched for miRNAs at the acute stage, (B) – pathways enriched for miRNAs at the latent stage, (C) – pathways enriched for miRNAs at the chronic stage; non-relevant pathways were excluded; FDR<0.05.

B

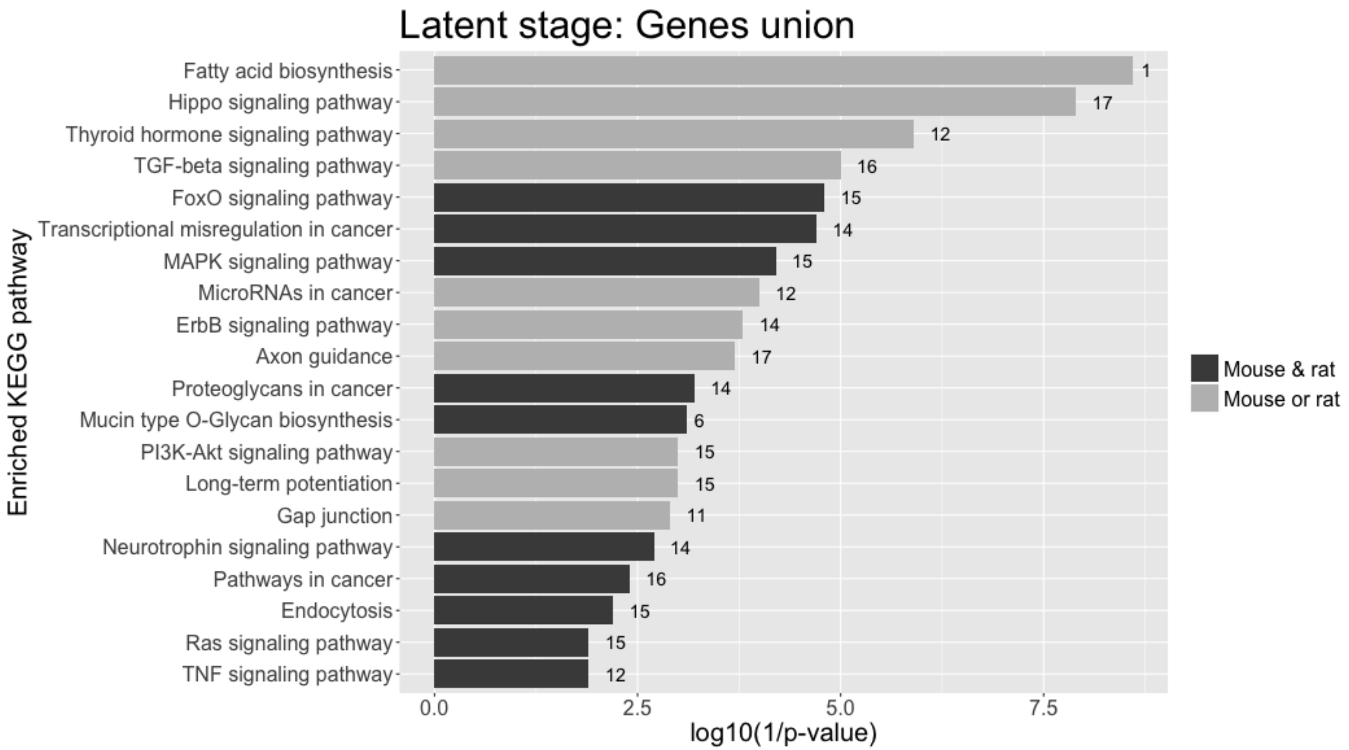

Supplementary Fig. S3: KEGG pathways enrichment analysis performed using DIANA miRPath ver.3 algorithm (Gene union mode) for consistently differentially expressed miRNAs, produced by RRA analysis and human TLE miRNAs. The first 10 most enriched pathways for each section are presented; (A) – Pathways enriched for miRNAs at the acute stage, (B) – pathways enriched for miRNAs at the latent stage, (C) – pathways enriched for miRNAs at the chronic stage; non-relevant pathways were excluded; FDR<0.05.

C

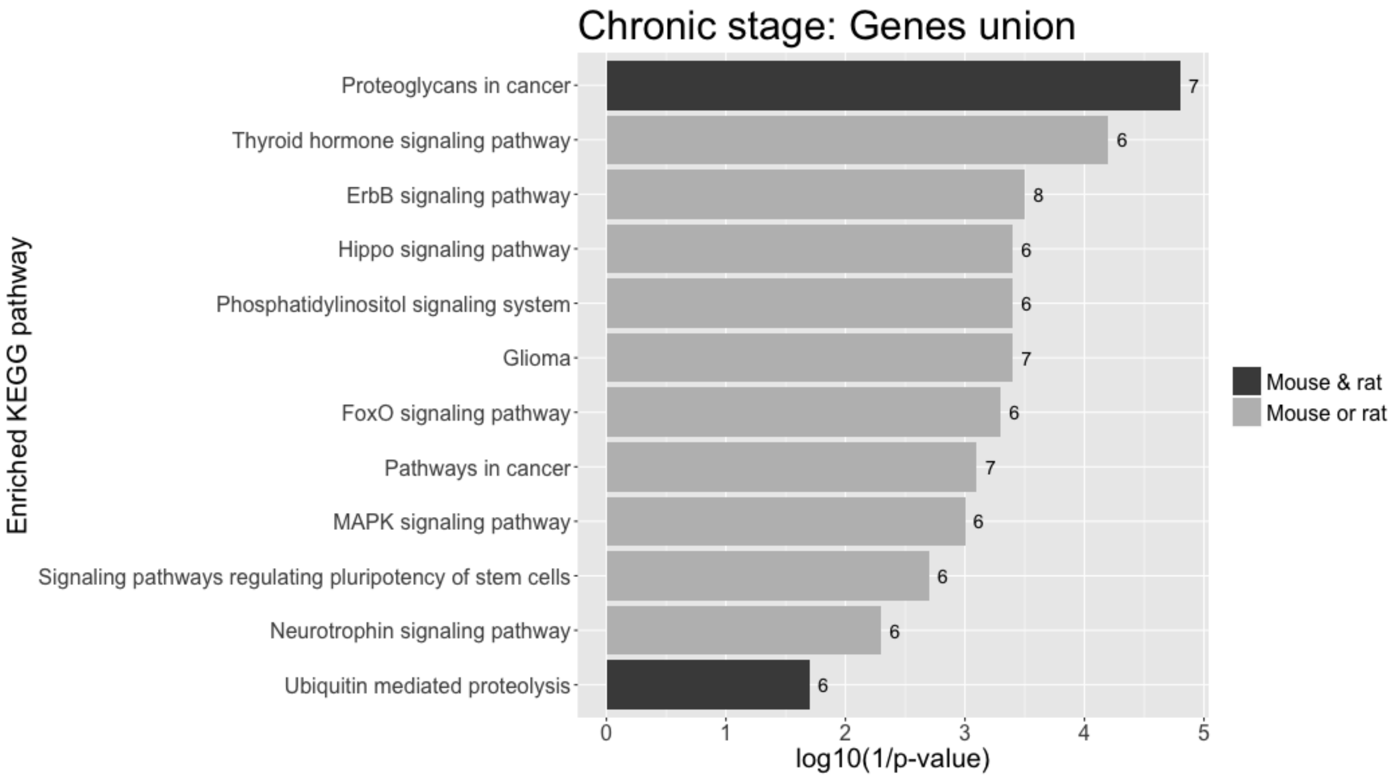

Supplementary Fig. S3: KEGG pathways enrichment analysis performed using DIANA miRPath ver.3 algorithm (Gene union mode) for consistently differentially expressed miRNAs, produced by RRA analysis and human TLE miRNAs. The first 10 most enriched pathways for each section are presented; (A) – Pathways enriched for miRNAs at the acute stage, (B) – pathways enriched for miRNAs at the latent stage, (C) – pathways enriched for miRNAs at the chronic stage; non-relevant pathways were excluded; FDR<0.05.

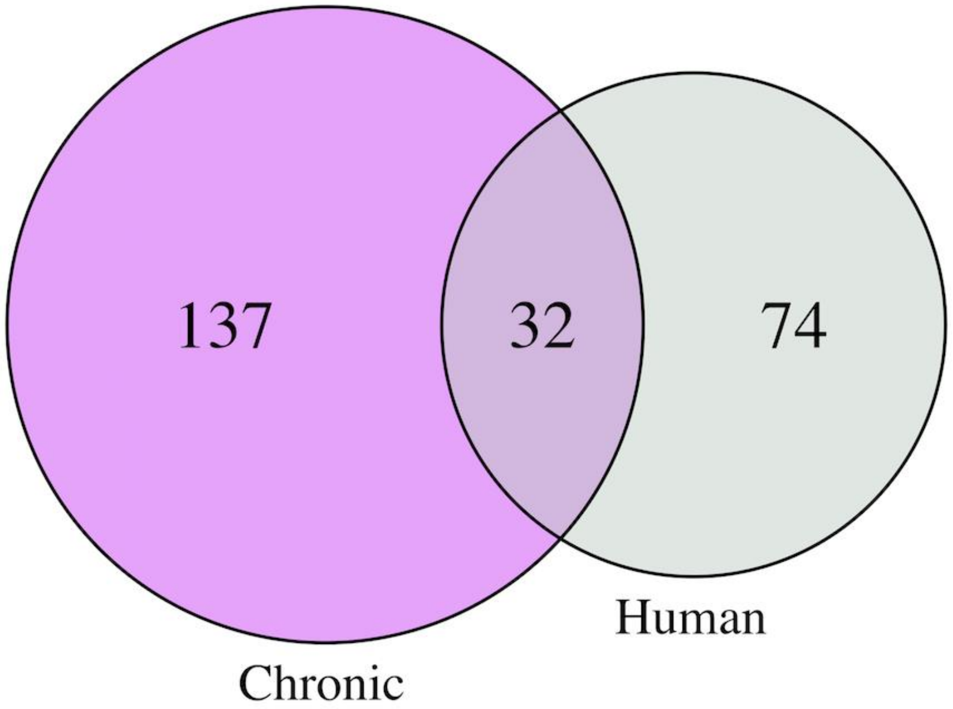

Supplementary Fig. S4: The overlap between the differentially expressed miRNAs in TLE-HS and chronic post-SE profiles. About 30% of miRNAs were shared between the 2 groups.

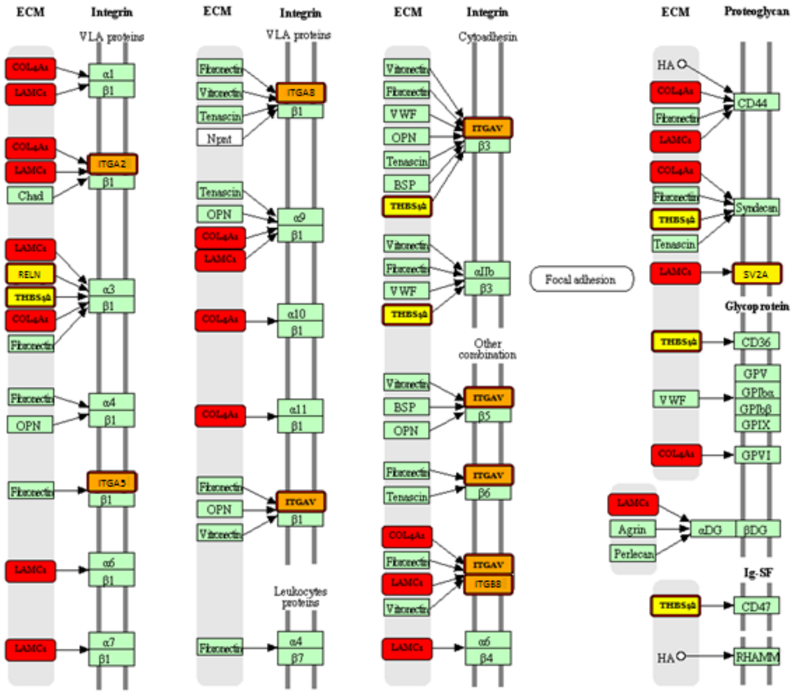

miRNAs associated with the ECM-receptor interaction pathway	
TLE-HS miRNAs	Chronic post-SE miRNAs
32-5p	346-5p
182-5p	92b-3p
376c-3p	98-5p
10b-5p	322-5p
27a-3p	370-5p
92b-3p	467c-3p
let-7b-5p	467e-3p
642a-5p	150-5p
29c-3p	467d-3p
27b-3p	137-3p
211-5p	466f-3p
665	669f-3p
let-7d-5p	152-3p
92a-3p	34c-5p
204-5p	29b-3p
	29a-3p
	146a-5p

Supplementary Fig. S5: The example map of genes targeted within the “ECM-receptor interaction” pathway by the miRNAs in TLE-HS profiles. The number of genes were predicted to be targeted by the miRNAs in both TLE-HS and chronic post-SE datasets (highlighted: red – collagens and laminins, orange – integrins, yellow – other genes). Only miR-92b-3p (highlighted in purple) was common for the two datasets; this figure was adapted from the diagram generated by Diana mirPath v.3 web tool [69] with the use of KEGG pathways [77-79].

Supplementary Table S1: the list of studies (extended table 1)

A: Included post-SE studies

Study	Injury model	Species	Platform	Profiled (changed)	Cut-off criteria	Time points	Region	Drug history	Notes	RNA extraction
Liu et al. (2010)	KA	Rats (n=6)	TaqMan miRNA assays (Applied Biosystems)	381 (10)	> 1.5-fold, FDR<0.05	24h	HP	KA (10mg/kg, s.c.), ISO	Brain ischemia and hemorrhage were also investigated in this study. Profile in blood was done	miRNeasy Mini Kit (Qiagen)
Hu et al. (2011)	PILO	Rats (n=6)	Rat MicroRNA array (Signosis, USA)	113 (26)	>2-fold, p<0.05	24h	HP	2X Chloral hydrate (10%, 5mL/kg), LiCl (125mg/kg, i.p, pilocarpine 20mg/kg i.p.), pilocarpine (20mg/kg i.p.) + adds	qPCR for 4 miRs was done in blood	Animal Tissue RNA Purification Kit (Norgen, Canada)
Song et al. (2011)	PILO	Rats (n=3)	Microarray (µParaflo, LC Sciences)	349 (23)	>1-fold (at least 2 of 3 probes >1.5-fold), p<0.01	60d	HP	atropine (i.p., 1 mg/kg), diazepam (i.p., 10mg/kg),lithium/pilocarpine (10mg/kg, i.p.) + adds	The time between the last spontaneous seizure and the time the animals were sacrificed was < 5h	Trizol Reagent (Invitrogen)
Jimenez-Mateos et al. (2011**)**	KA	Mice	TaqMan low-density arrays (TLDA) MicroRNA assays ver.1.0 (Applied Biosystems)	380 (29)	>1.5-fold, p<0.05	24h	CA3	ISO 5%, kainic acid (i.ag.), lorazepam (Ativan; 6 mg/kg, i.p.), pentobarbital overdose	Preconditioning was studied	miRNeasy kit (Qiagen)
Hu et al. (2012)	PILO	Rats (n=6)	Microarray (Agilent)	350 (24)	>1.5-fold, p<0.05	60d	HP	chloral hydrate (10%, 3 ml/kg, i.p.), LiCl (125 mg/kg i.p.), pilocarpine (20 mg/kg, i.p.) + adds		mirVana (Ambion)
Bot et al. (2013)	ES (amygdala)	Rats (n=5)	Microarray miRCURY LNA 7 (Exiqon)	3100/292 (66)	P<0.05, FDR	7d, 14d, 30d, 90d	DG	ISO 2-2.5%, i.p. butorphanol (0.5 mg/kg i.p.), diazepam (20 mg/kg)+adds	EEG monitoring showed occurence of seizures at 7 days and more seizures at 14 days. 14 days and 90 days time points have been excluded, because there was no difference in miRNA expression	miRNeasy Mini kit (QIAGEN)
Sun et al. (2013)	ES (amygdala)	Rats (n=6)	Sequencing Illumina HiSeq 2000 (Illumina)	All (6)	≥30 copies, >2-fold, P<0.01	24h	HP	10% chloral hydrate (3.5 mL/kg, i.p.)		mirVana miRNA Isolation Kit (Ambion)
Li et al. (2014)	ES (amygdala)	Rats (n=6)	Sequencing (Illumina)	All (25)	≥10 copies, >1.5-fold, FDR <0.05	2m	HP	10% chloral hydrate (3.5 mL/kg, i.p.)		mirVana miRNA Isolation Kit (Ambion)
Gorter et al. (2014)	ES (angular bundle)	Rats (n=5-6)	Microarray miRCURY LNA 6 (Exiqon)	2383/322 (DG-37, CA1-7)	>1.5-fold, p<0.01, FDR	24h, 7d, 3-4m	DG, CA1	ketamine (57 mg/kg, i.m.), xylazine (9 mg/kg, i.m.)	3 miRs were measured in plasma by qPCR	miRNeasy kit (Qiagen Benelux)
Krestschmann et al. (2015)	ES (amygdala)	Mice (n=8)	Microarray miRCURY LNA 5 (Exiqon)	579 (24h-91, 28d-60)	>1-fold, FDRBH<0.05	24h, 28d	HP	diazepam (10 mg/kg, i.p.)	Both ES and pilocarpine models were used and compared in this study	miRVana miRNA isolation kit (Ambion)
Krestschmann et al. (2015)	PILO	Mice (n=8)	Microarray miRCURY LNA 5 (Exiqon)	579 (24h-91, 28d-60)	>1-fold, FDRBH<0.05	24h, 28d	HP	N-methylscopolamine (1 mg/kg (i.p.), pilocarpine (300 mg/kg, i.p.), diazepam (10 mg/kg, i.p.)	Both E/S and pilocarpine models were used and compared in this study	miRVana miRNA isolation kit (Ambion)
Schouten et al. (2015)	KA	Mice (n=3)	TaqMan Mouse MicroRNA Fluidic v3.0 Cards	381 (189)	>1.5-fold, FDR<0.05	72h	DG	KA (24mg/kg)+adds		Trizol reagent (Invitrogen)

B. Included human TLE-HS studies

Study	Epilepsy type	Platform	Profiled (regulated)	Criteria	Region	Drug history	Notes	RNA extraction
Kan et al. (2012)	TLE-HS (n=10)	miRCURY LNA Array 5 (Exiqon)	130 (51)	>2-fold, P< 3.92 x 10^-5 (Bonferroni)	HP	Various AEDs	Only the group with HS included	miRNeasy kit (Qiagen)
McKiernan et al. (2012)	TLE-HS (n=11)	TLDA v1.0 (Applied Biosystems)	380 (47)	>1.5-fold, p<0.05	HP&TC	Various AEDs	8 hippocampal samples mixed with 3 neocortical samples	miRNeasy kit (Qiagen)
Kaalund et al. (2014)	TLE-HS (n=10)	miRCURY LNA array 7.1 (Exiqon)	(30)	SAM (FDR = 23.69, delta 0.42)	HP	Various AEDs	Only HS group included	TRIzol (Invitrogen)

C: Not included post-SE studies

Study	Injury model	Species	Platform	Profiled (changed)	Cut-off criteria	Time points	Region	Drug history	Notes	RNA extraction
Risbud& Porter (2012)	PILO	Rats	Microarray (Exiqon 10.2)		p<0.05	4h, 48h, 3w	HP	ISO, methyl-scopolamine (1 mg/kg, i.p.)/pilocarpine (385 mg/kg, i.p.) +adds, valium 6 mg/kg	Controls received 1/10 of experimental pilocarpine dose	mirVANA miRNA isolation kit (Ambion)
McKiernan et al. (2012)	KA	Mice (n=4)	TaqMan TLDA MicroRNA assays version 1.0 (Applied Biosystems)	380	>1.5-fold, p<0.05	24h	CA3	ISO, kainic acid, lorazepam 6 mg/kg, i.p.). pentobarbital overdose	Experimental group received preconditioning stimuli	miRNA easy kit (Qiagen)

D: Not included TLE studies

Study	Epilepsy type	Platform	Profiled (regulated)	Criteria	Region	Drug history	Notes	RNA extraction
Haenisch et al. (2015)	TLE (n=8)	TLDA MicroRNA A+B Cards Set v3.0 (Applied Biosystems)	754 (16)	>2-fold, p<0.05	HP&TC	Various AEDs	Non-focal tissue was used as control	mirVana PARIS Kit (Ambion)
Danis et al. (2016)	TLE-HS (n=14)	Illumina deep sequencing (Vertis Biotechnologie AG)	894 (1)	FDR<0.01	HP	Various AEDs	HS tissue compared with non-HS tissue without control	mirVana (Life Technologies)

Supplementary Table 1: (A) **-** miRNA profiling studies in animal post-SE models that passed the criteria for inclusion in the meta-analysis. The SE in these animal models was induced by chemoconvulsants kainic acid (KA) and pilocarpine (PILO) or by electrical stimulation of a selected brain area. The two species most often used were rats and mice. Cut-off criteria for differential expression of miRNAs were kept as reported by the authors of each study and were based on the threshold of deregulation (expressed as fold-change) and the p-value of differential expression (adjusted or non-adjusted). Various time-points were used as indicated (h - hours, d - days, w- weeks, m – months following SE). Those profiling studies were included where the whole hippocampus (HP) or related regions (DG – dentate gyrus, GCL – granule cell layer of the DG, CA – Cornu Ammonis) were investigated; (B) - included studies in human temporal lobe epilepsy with hippocampal sclerosis (TLE-HS); (C) – not included animal studies; (D) – not included studies in TLE. Other abbreviations: TC – temporal cortex, ISO – isoflurane, i.ag. – intra-amygdalar, i.p. – intraperitoneal, BH - Benjamini-Hochberg, SAM - significance analysis of microarrays, AED - anti-epileptic drug, CCI – controlled cortical impact, CV - coefficient of variation, SD - standard deviation

Supplementary Tables S2-S6 are available as spreadsheets

Supplementary Table S7
A. Pathway enrichment analysis for 100 miRNAs from post-SE rodent profiles obtained during the chronic stage.

KEGG pathway	p-value	N genes	N miRNAs
ECM-receptor interaction	6.69E-110	32	16
Fatty acid biosynthesis	1.45E-23	3	3
Drug metabolism - cytochrome P450	3.51E-16	10	2
Fatty acid degradation	4.86E-16	5	5
Mucin type O-Glycan biosynthesis	4.25E-14	11	13
Fatty acid metabolism	2.96E-12	7	8
Biosynthesis of unsaturated fatty acids	3.06E-11	3	6
Glycosphingolipid biosynthesis - lacto and neolacto series	5.47E-11	7	19
Thyroid hormone synthesis	1.57E-09	8	5
GABAergic synapse	2.84E-09	34	17
Morphine addiction	1.33E-08	23	9
Protein digestion and absorption	3.48E-08	19	3
Gap junction	4.30E-08	9	6
Hippo signaling pathway	4.30E-08	30	10
Proteoglycans in cancer	4.80E-08	90	19
Focal adhesion	9.54E-08	45	4
Proteasome	2.12E-07	2	1
Estrogen signaling pathway	2.15E-07	31	11
Glycosaminoglycan biosynthesis - heparan sulfate / heparin	3.15E-07	9	9
Arachidonic acid metabolism	7.51E-07	5	2
Transcriptional misregulation in cancer	1.02E-06	40	12
Ascorbate and aldarate metabolism	1.63E-06	8	1
ErbB signaling pathway	1.69E-06	25	7
Thyroid hormone signaling pathway	2.78E-06	45	10
Long-term depression	2.95E-06	14	7
Glioma	3.82E-06	28	16
Synaptic vesicle cycle	4.28E-06	3	2
PI3K-Akt signaling pathway	4.73E-06	68	5
Metabolic pathways	8.96E-06	5	1
Cell adhesion molecules (CAMs)	1.18E-05	23	7
Pentose and glucuronate interconversions	1.99E-05	8	1
Glycosaminoglycan biosynthesis - keratan sulfate	2.84E-05	4	4
N-Glycan biosynthesis	2.84E-05	9	6
Pathways in cancer	2.95E-05	70	7
Signaling pathways regulating pluripotency of stem cells	3.24E-05	57	17
Tyrosine metabolism	3.55E-05	1	2
Prolactin signaling pathway	3.86E-05	25	8
TGF-beta signaling pathway	3.99E-05	21	5
Phosphatidylinositol signaling system	4.33E-05	22	11
Cyanoamino acid metabolism	6.49E-05	1	1

Glutathione metabolism	6.49E-05	1	1
Galactose metabolism	8.84E-05	2	1
Other types of O-glycan biosynthesis	1.07E-04	1	1
Endocrine and other factor-regulated calcium reabsorption	1.10E-04	10	4
Glutamatergic synapse	1.10E-04	19	3
Wnt signaling pathway	1.49E-04	26	7
Lysine degradation	1.65E-04	11	6
Amino sugar and nucleotide sugar metabolism	1.98E-04	6	2
Circadian entrainment	2.27E-04	15	4
Endocytosis	2.34E-04	30	5
Nicotine addiction	2.71E-04	15	11
Cytokine-cytokine receptor interaction	3.08E-04	15	4
leucine and isoleucine degradation	3.71E-04	1	1
Chemical carcinogenesis	4.28E-04	10	2
Amphetamine addiction	4.53E-04	28	11
Axon guidance	5.37E-04	34	5
Starch and sucrose metabolism	5.43E-04	8	1
NF-kappa B signaling pathway	6.79E-04	9	2
Fatty acid elongation	7.58E-04	1	2
Glycosaminoglycan degradation	7.61E-04	3	2
Protein processing in endoplasmic reticulum	8.73E-04	24	4
Long-term potentiation	1.02E-03	22	5
Adrenergic signaling in cardiomyocytes	1.82E-03	37	6
D-Glutamine and D-glutamate metabolism	1.84E-03	2	2
Taurine and hypotaurine metabolism	1.96E-03	3	2
Acute myeloid leukemia	2.04E-03	16	5
Retrograde endocannabinoid signaling	2.08E-03	6	1
FoxO signaling pathway	2.37E-03	33	6
Thyroid cancer	2.37E-03	8	7
Regulation of actin cytoskeleton	2.66E-03	37	4
Glycosylphosphatidylinositol(GPI)-anchor biosynthesis	2.67E-03	5	4
Aspartate and glutamate metabolism	2.73E-03	3	4
MAPK signaling pathway	2.85E-03	45	4
Oxytocin signaling pathway	2.85E-03	33	4
Rap1 signaling pathway	2.85E-03	50	4
Glycerophospholipid metabolism	3.18E-03	9	1
D-Arginine and D-ornithine metabolism	3.24E-03	1	1
Phagosome	3.91E-03	1	1
alpha-Linolenic acid metabolism	3.97E-03	1	2
Vascular smooth muscle contraction	4.01E-03	19	3
Colorectal cancer	4.15E-03	19	6
Renal cell carcinoma	4.94E-03	9	3
Notch signaling pathway	4.96E-03	8	3
Adherens junction	5.86E-03	12	4

MicroRNAs in cancer	5.97E-03	14	4
Ubiquitin mediated proteolysis	6.12E-03	32	4
Inflammatory mediator regulation of TRP channels	6.49E-03	9	1
Toll-like receptor signaling pathway	6.50E-03	4	1
Insulin signaling pathway	6.69E-03	27	3
Lysosome	7.71E-03	1	1
Central carbon metabolism in cancer	7.91E-03	5	3
ABC transporters	8.31E-03	5	1
Circadian rhythm	8.69E-03	17	7
Linoleic acid metabolism	9.13E-03	4	2
Tight junction	9.13E-03	8	2
Cocaine addiction	9.43E-03	1	1
Nicotinate and nicotinamide metabolism	9.46E-03	4	1
Glycosphingolipid biosynthesis - globo series	1.00E-02	2	1
Ribosome biogenesis in eukaryotes	1.00E-02	2	1
RNA degradation	1.01E-02	1	1
Proximal tubule bicarbonate reclamation	1.02E-02	4	1
AMPK signaling pathway	1.06E-02	8	2
Glycosaminoglycan biosynthesis - chondroitin sulfate / dermatan sulfate	1.08E-02	1	2
Neurotrophin signaling pathway	1.23E-02	28	6
Regulation of autophagy	1.36E-02	1	1
mTOR signaling pathway	1.39E-02	18	6
Serotonergic synapse	1.51E-02	6	2
Selenocompound metabolism	1.53E-02	2	1
cGMP-PKG signaling pathway	1.53E-02	33	4
Sulfur metabolism	1.61E-02	1	1
Ras signaling pathway	1.66E-02	9	2
Steroid biosynthesis	1.66E-02	1	2
Dopaminergic synapse	1.75E-02	9	1
Hedgehog signaling pathway	2.03E-02	7	2
Sphingolipid metabolism	2.10E-02	4	3
T cell receptor signaling pathway	2.27E-02	22	4
VEGF signaling pathway	2.27E-02	8	1
Inositol phosphate metabolism	2.29E-02	8	3
Mismatch repair	2.73E-02	2	1
Tryptophan metabolism	2.81E-02	1	1
Propanoate metabolism	2.81E-02	1	1
Glycosphingolipid biosynthesis - ganglio series	2.99E-02	1	1
Degradation of aromatic compounds	3.13E-02	1	1
Choline metabolism in cancer	3.24E-02	2	1
Neuroactive ligand-receptor interaction	3.69E-02	6	1
Oxidative phosphorylation	3.87E-02	1	1
Butanoate metabolism	4.01E-02	1	1
Homologous recombination	4.05E-02	1	1

leucine and isoleucine biosynthesis	4.14E-02	1	1
Calcium signaling pathway	4.18E-02	9	1
Vitamin B6 metabolism	4.32E-02	1	1
Folate biosynthesis	4.75E-02	1	1
HIF-1 signaling pathway	4.79E-02	5	2
cAMP signaling pathway	4.85E-02	16	1

B. Pathway enrichment analysis for 100 miRNAs from TLE-HS profiles

KEGG pathway	p-value	N genes	N miRNAs
ECM-receptor interaction	6.21E-124	43	15
Fatty acid biosynthesis	2.93E-39	5	4
Fatty acid metabolism	7.06E-18	19	9
Biosynthesis of unsaturated fatty acids	6.19E-17	9	7
Thyroid hormone synthesis	1.35E-14	5	6
Mucin type O-Glycan biosynthesis	2.94E-14	18	17
Glycosphingolipid biosynthesis - lacto and neolacto series	8.24E-12	13	12
Protein digestion and absorption	4.28E-11	26	2
TGF-beta signaling pathway	2.57E-10	60	22
Morphine addiction	3.53E-09	56	18
Biotin metabolism	4.57E-09	1	3
Lysine degradation	2.58E-08	27	14
Signaling pathways regulating pluripotency of stem cells	1.65E-07	96	22
Hippo signaling pathway	1.89E-07	81	14
Glycosaminoglycan biosynthesis - heparan sulfate / heparin	2.56E-07	10	10
Fatty acid degradation	7.57E-07	3	3
Focal adhesion	8.68E-07	111	9
Axon guidance	8.69E-06	90	18
Cytokine-cytokine receptor interaction	1.44E-05	32	3
PI3K-Akt signaling pathway	1.69E-05	171	10
Wnt signaling pathway	1.82E-05	59	10
Amphetamine addiction	1.95E-05	36	14
GABAergic synapse	2.39E-05	36	9
Fatty acid elongation	2.81E-05	4	2
Thyroid hormone signaling pathway	3.32E-05	48	10
Pathways in cancer	3.54E-05	181	11
ErbB signaling pathway	4.33E-05	50	10
Endocytosis	5.45E-05	48	4
Glioma	8.39E-05	41	18
Estrogen signaling pathway	8.85E-05	42	12
Proteoglycans in cancer	1.04E-04	130	23
Metabolism of xenobiotics by cytochrome P450	1.79E-04	8	4
Glycosphingolipid biosynthesis - ganglio series	2.53E-04	7	5
Regulation of actin cytoskeleton	2.95E-04	98	11
Protein processing in endoplasmic reticulum	3.30E-04	39	5
cGMP-PKG signaling pathway	3.64E-04	39	5

Phosphatidylinositol signaling system	3.71E-04	35	7
Neurotrophin signaling pathway	3.93E-04	40	5
FoxO signaling pathway	4.72E-04	78	15
Long-term depression	5.54E-04	36	11
Glycosaminoglycan biosynthesis - keratan sulfate	6.53E-04	7	7
Synaptic vesicle cycle	6.92E-04	12	3
Antigen processing and presentation	8.28E-04	2	1
N-Glycan biosynthesis	8.41E-04	11	7
Glycosaminoglycan biosynthesis - chondroitin sulfate / dermatan sulfate	1.27E-03	6	4
Cocaine addiction	1.41E-03	23	10
MAPK signaling pathway	1.53E-03	61	5
Rap1 signaling pathway	1.82E-03	74	5
p53 signaling pathway	2.09E-03	28	7
Gap junction	2.13E-03	27	7
RNA degradation	2.48E-03	1	1
Transcriptional misregulation in cancer	3.18E-03	51	3
One carbon pool by folate	3.26E-03	6	1
Nicotinate and nicotinamide metabolism	3.73E-03	4	3
Drug metabolism - cytochrome P450	3.78E-03	8	2
Porphyrin and chlorophyll metabolism	3.78E-03	10	2
AMPK signaling pathway	3.84E-03	44	8
Ubiquinone and other terpenoid-quinone biosynthesis	4.63E-03	1	1
MicroRNAs in cancer	5.23E-03	43	6
Ubiquitin mediated proteolysis	5.63E-03	49	7
Lysine biosynthesis	5.73E-03	1	1
Thyroid cancer	5.99E-03	9	4
Glycosylphosphatidylinositol(GPI)-anchor biosynthesis	7.10E-03	3	2
Adherens junction	7.15E-03	22	5
Cell adhesion molecules (CAMs)	7.45E-03	29	5
Leucine and isoleucine degradation	7.55E-03	5	3
Inositol phosphate metabolism	8.50E-03	22	3
Insulin signaling pathway	8.82E-03	26	3
Amino sugar and nucleotide sugar metabolism	9.22E-03	5	2
Glutamatergic synapse	1.01E-02	31	5
Tryptophan metabolism	1.15E-02	5	1
Glycerophospholipid metabolism	1.16E-02	33	4
mTOR signaling pathway	1.20E-02	22	5
Inflammatory mediator regulation of TRP channels	1.26E-02	10	1
Ascorbate and aldarate metabolism	1.27E-02	7	2
Nicotine addiction	1.27E-02	20	5
Amyotrophic lateral sclerosis (ALS)	1.40E-02	13	4
Retrograde endocannabinoid signaling	1.42E-02	23	3
Choline metabolism in cancer	1.46E-02	19	2
Ras signaling pathway	1.59E-02	90	6

Sphingolipid metabolism	1.65E-02	8	2
Circadian rhythm	1.66E-02	16	7
Hedgehog signaling pathway	1.73E-02	13	2
Leucine and isoleucine biosynthesis	2.15E-02	1	4
Cysteine and methionine metabolism	2.15E-02	1	1
Pentose and glucuronate interconversions	2.15E-02	1	1
Pyruvate metabolism	2.15E-02	1	1
Citrate cycle (TCA cycle)	2.15E-02	1	1
Glyoxylate and dicarboxylate metabolism	2.15E-02	1	1
Homologous recombination	2.21E-02	1	1
Dopaminergic synapse	2.32E-02	19	3
Long-term potentiation	2.35E-02	30	6
2-Oxocarboxylic acid metabolism	2.53E-02	2	2
SNARE interactions in vesicular transport	2.53E-02	2	1
Sphingolipid signaling pathway	2.69E-02	1	1
Cholinergic synapse	2.81E-02	11	1
D-Arginine and D-ornithine metabolism	2.92E-02	1	2
Huntington's disease	3.07E-02	10	1
GnRH signaling pathway	3.09E-02	4	1
Renin-angiotensin system	3.49E-02	5	1
Serotonergic synapse	3.66E-02	10	1
Insulin secretion	3.70E-02	1	1
TNF signaling pathway	3.71E-02	5	1
Tight junction	3.73E-02	1	1
Carbon metabolism	3.75E-02	1	1
Metabolic pathways	3.75E-02	2	1
Vitamin digestion and absorption	4.02E-02	2	1
Jak-STAT signaling pathway	4.09E-02	11	2
Cell cycle	4.43E-02	1	1
Central carbon metabolism in cancer	4.47E-02	9	1
RNA transport	4.75E-02	10	1

Supplementary Table S7: The KEGG pathway enrichment analysis for: (**A**) 100 miRNAs most consistently differentially expressed in post-SE rodent profiles obtained during the chronic stage; (**B**) for 100 miRNAs most consistently differentially expressed in human TLE-HS profiles; The analysis was done using DIANA miRPath v.3 using “Pathway Union” mode and demonstrated the enriched pathways associated with the miRNAs that were found in chronic post-SE profiles and TLE-HS profiles along with the number of genes predicted to be regulated within these pathways; FDR<0.05
